# Annexin A1 attenuates cardiac diastolic dysfunction in mice with inflammatory arthritis

**DOI:** 10.1073/pnas.2020385118

**Published:** 2021-09-15

**Authors:** Jianmin Chen, Lucy V. Norling, Jose Garrido Mesa, Marina De Paula Silva, Sophie E. Burton, Chris Reutelingsperger, Mauro Perretti, Dianne Cooper

**Affiliations:** ^a^William Harvey Research Institute, Barts and The London School of Medicine and Dentistry, Queen Mary University of London, London EC1M 6BQ, United Kingdom;; ^b^Department of Biochemistry, Cardiovascular Research Institute, Maastricht University, Maastricht 6229HX, The Netherlands

**Keywords:** cardiomyopathy, diastolic dysfunction, HFpEF, arthritis, annexin A1

## Abstract

Rheumatoid arthritis (RA) carries a twofold increased incidence of heart failure with preserved ejection fraction, accompanied by diastolic dysfunction, which can lead to death. The causes of diastolic dysfunction are unknown, and there are currently no well-characterized animal models for studying these mechanisms. Current medications for RA do not have marked beneficial cardio-protective effects. K/BxN F1 progeny and KRN control mice were analyzed over time for arthritis development, monitoring left ventricular diastolic and systolic function using echocardiography. Excised hearts were analyzed by flow cytometry, qPCR, and histology. In pharmacological experiments, K/BxN F1 mice were treated with human recombinant AnxA1 (hrAnxA1, 1 μg/mouse) or vehicle daily. K/BxN F1 mice exhibited fully developed arthritis with normal cardiac function at 4 wk; however, by week 8, all mice displayed left ventricular diastolic dysfunction with preserved ejection fraction. This dysfunction was associated with cardiac hypertrophy, myocardial inflammation and fibrosis, and inflammatory markers. Daily treatment of K/BxN F1 mice with hrAnxA1 from weeks 4 to 8 halted progression of the diastolic dysfunction. The treatment reduced cardiac transcripts of proinflammatory cytokines and profibrotic markers. At the cellular level, hrAnxA1 decreased activated T cells and increased MHC II^low^ macrophage infiltration in K/BxN F1 hearts. Similar effects were obtained when hrAnxA1 was administered from week 8 to week 15. We describe an animal model of inflammatory arthritis that recapitulates the cardiomyopathy of RA. Treatment with hrAnxA1 after disease onset corrected the diastolic dysfunction through modulation of both fibroblast and inflammatory cell phenotype within the heart.

## Significance

Patients with rheumatoid arthritis (RA) are susceptible to heart failure accompanied by diastolic dysfunction. It is unknown what causes diastolic dysfunction in RA, and current therapies do not reduce the risk of heart problems. Here, we characterize a murine model of arthritis, which mirrors the diastolic dysfunction observed in RA patients; this dysfunction is associated with fibrous tissue formation, enlargement of the heart, and changes in the number/type of inflammatory cells within the heart. Importantly, treatment with the protein Annexin A1 not only halts the progression of diastolic dysfunction, but, when given at a later stage, it reverses established diastolic dysfunction and attenuates cardiac remodeling in arthritic mice. This model can help develop treatments for heart failure in RA.

Rheumatoid arthritis (RA) is a chronic inflammatory condition of the joints that causes pain, reduced mobility, and impaired quality of life. While the joint disease per se is not lethal, around half of all premature deaths in RA patients are due to cardiovascular comorbidities (1, 2), among which nonischemic heart failure plays a predominant role (3–5), although the pathogenesis of nonischemic heart failure may include undetected coronary artery disease (6). The incidence of congestive heart failure (CHF) in patients with RA is approximately twofold higher than the general population (7). RA patients are particularly susceptible to heart failure with preserved ejection fraction (HFpEF), which is accompanied by a diastolic dysfunction with normal or near normal EF (8). Even without clinical evidence of heart malfunction, around 40% of RA patients present asymptomatic left ventricular (LV) diastolic dysfunction with or without preserved systolic function. This diastolic dysfunction, if progressive over time, contributes to CHF (9). While 80% of CHF in the general population is attributed to traditional risk factors (hyperlipidemia, diabetes, and hypertension), in RA these factors are linked to only 40% of CHF incidence (10). Therefore, there are unexplained risk factor(s) for CHF in RA patients likely related to systemic and local inflammation; however, these mechanism(s) remain by and large elusive. Moreover, limited beneficial cardio-protective effects have been observed for medications currently used in RA treatment, while in some cases, deterioration has been reported (5): the clinical unmet need for increased CHF in RA remains pressing.

While acute inflammation is a protective reaction to invading pathogens or tissue damage, this protection is ideally contained in time and space. When inflammation is prolonged, it can expand from the primary tissue to secondary sites resulting in development/exacerbation of comorbidities like the enhanced cardiovascular disease observed in RA patients (11). Physiological inflammation actively resolves, governed by endogenous mediators signaling through G protein-coupled receptors (GPCRs), including ALX/FPR2. In fact, the formyl-peptide receptor (FPR) family of GPCRs plays an important role in host defense, regulation of inflammation, and its resolution (12). Particularly, FPR2 facilitates resolution of inflammation in experimental inflammatory arthritis upon activation by annexin A1 (AnxA1) (13) and resolvin D1 (14). Equally importantly, FPR2 agonists such as AnxA1 (15), resolvin D1 (16), and lipoxin A_4_ (17) are cardio protective in experimental settings of myocardial infarction. FPRs might therefore represent a therapeutic target for the cardiomyopathy associated with inflammatory arthritis.

To start elucidating mechanisms and potential therapeutic strategies, we have characterized cardiac function in a mouse model of arthritis, the K/BxN F1 progeny, recapitulating the diastolic dysfunction reported in RA patients. These mice express both the T cell receptor transgene KRN and the major histocompatibility complex (MHC) class II molecule A^g7^ from the NOD/ShiLtJ mouse strain leading to the development of arthritis spontaneously (18). Having established this clinically relevant model of diastolic dysfunction associated with arthritis, we then investigated the potential beneficial effects of human recombinant AnxA1 (hrAnxA1) against the diastolic dysfunction of K/BxN F1 mice.

## Results

### Arthritic Mice Develop Diastolic Dysfunction with Preserved EF.

**Fig. 1.**
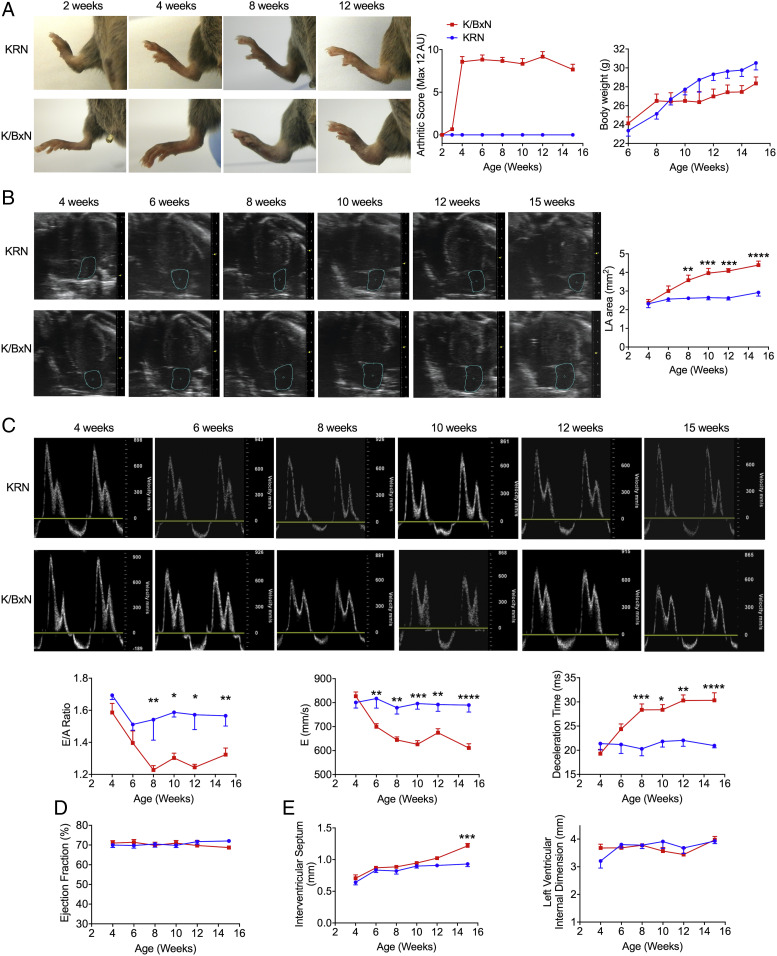
Arthritic K/BxN F1 mice display diastolic dysfunction but normal systolic function as assessed by echocardiography. (*A*) K/BxN F1 mice develop marked arthritis from week 4 with no significant change in body weight. Clinical severity of arthritis was assessed visually using a defined severity scoring system (max score 12). (*B* and *C*) K/BxN F1 mice fully develop diastolic dysfunction from week 8. (*B*) Representative B-mode four-chamber echocardiograms and increased LA area in arthritic mice. (*C*) Representative mitral flow patterns from pulsed-wave color Doppler echocardiography, decreased E/A ratio, decreased E wave velocity, and increased deceleration time in arthritic mice. (*D*) K/BxN F1 mice show no significant change in percentages of EF using M-mode echocardiograms. (*E*) K/BxN F1 mice develop concentric cardiac hypertrophy at week-15 indicated by an increase in interventricular septum thickness with no difference in LV internal diastolic dimensions. Data are mean ± SEM. KRN group: *n* = 5; K/BxN F1 group: *n* = 6. *P < 0.05, **P < 0.01, ***P < 0.001, ****P < 0.0001 versus nonarthritic KRN group (two-way ANOVA followed by Bonferroni’s test). K/BxN: K/BxN F1.

In our quest for a model of inflammatory arthritis which could display HFpEF and diastolic cardiomyopathy, we tested the F1 progeny of the KRN mice coupled to the NOD colony (18). In initial experiments, ankle swelling first became visible from week 3 and plateaued from week 4 with near maximal clinical score (Fig. 1*A*). Control KRN mice did not show any indication of arthritis. There was a nonsignificant trend of reduced body weight in K/BxN F1 mice compared to KRN mice from 10 wk onwards (Fig. 1*A*). The same groups were followed for the development of cardiac dysfunction using echocardiography. When compared with KRN mice, enlargement of left atrial (LA) area, one of the most sensitive markers for diastolic dysfunction (19), was first detectable in half of K/BxN F1 mice at week 6 (Fig. 1*B*). By week 8, all K/BxN F1 mice showed enlarged LA area, reduced E/A ratio, reduced E wave, and increased deceleration time (Fig. 1 *B* and *C*), indicating full development of diastolic dysfunction. Enlargement of LA area and increased deceleration time deteriorated further over time and were monitored until week 15 (Fig. 1 *B* and *C*). It is noteworthy that EF, as an indicator of systolic function, was preserved in K/BxN F1 mice throughout the entire time-course (Fig. 1*D*).

In line with previous work (20), we observed a degree of mitral valve inflammation in 15-wk-old K/BxN F1 mice, indicated by thickened mitral valve leaflets (*SI Appendix*, Fig. S1). Functional analysis of the mitral valve using color Doppler showed that only two out of 10 15-wk-old K/BxN F1 mice developed trivial mitral regurgitation, indicated by a blue jet area in LA as the mitral valve closes at the early stage of systolic phase (Video S1), while the rest of K/BxN F1 mice did not show signs of mitral regurgitation (Video S2). Trivial mitral regurgitation was also observed in age-matched KRN mice (Video S3), indicating that this phenomenon may occur within normal physiological settings. These findings indicate that the LV diastolic dysfunction in K/BxN F1 mice was not secondary to mitral regurgitation. No obvious mitral stenosis was observed in 15-wk-old K/BxN F1 mice as indicated by similar pattern/width of color jet in the LV during diastolic phase (Video S2) to that observed in age-matched KRN mice (Video S3). In these mice, we did not observe evidence of pericardial (Video S4) or pleural fluid accumulation (Video S5), making it highly unlikely that the LV diastolic dysfunction observed in K/BxN F1 mice could arise from these phenomena.

Additionally, we observed an increased lung wet to dry weight ratio in 8-wk-old K/BxN F1 mice compared with age-matched KRN mice (*SI Appendix*, Fig. S2*A*). The same trend was also observed in 15-wk-old K/BxN F1 mice in comparison with age-matched KRN mice (*SI Appendix*, Fig. S2*B*), indicating presence of pulmonary edema in K/BxN F1 mice. When monitoring physical activity, we observed that animal movement around the bottom of cage and rearing were normal in K/BxN F1 mice across different age groups. No unbalanced gait was seen. Speed of movement was slightly reduced in K/BxN F1 mice compared with KRN controls, with a reduced grip depending on the severity of joint disease. Moreover, there was no significant difference in mean arterial blood pressure, systolic pressure, diastolic pressure, or heart rate between 10-wk-old K/BxN F1 mice in comparison with age-matched KRN controls (*SI Appendix*, Fig. S2 *C–F*); the same observation was obtained in 15-wk-old K/BxN F1 mice in comparison with age-matched KRN mice (*SI Appendix*, Fig. S2 *G–J*), indicating that the LV diastolic dysfunction of arthritic mice, at least at weeks 10 and 15, was not secondary to alterations in blood pressure or heart rate.

Together, these data showed that arthritic K/BxN F1 mice developed diastolic dysfunction with preserved EF recapitulating the dysfunction reported in RA patients. The emergence of established diastolic dysfunction was 4 wk later than fully developed arthritis, thus defining two milestones in this mouse colony: week 4 (mice with arthritis but no diastolic dysfunction) and week 8 (mice with both arthritis and diastolic dysfunction).

### Cardiac Hypertrophy and Fibrosis in Arthritic Mice.

**Fig. 2.**
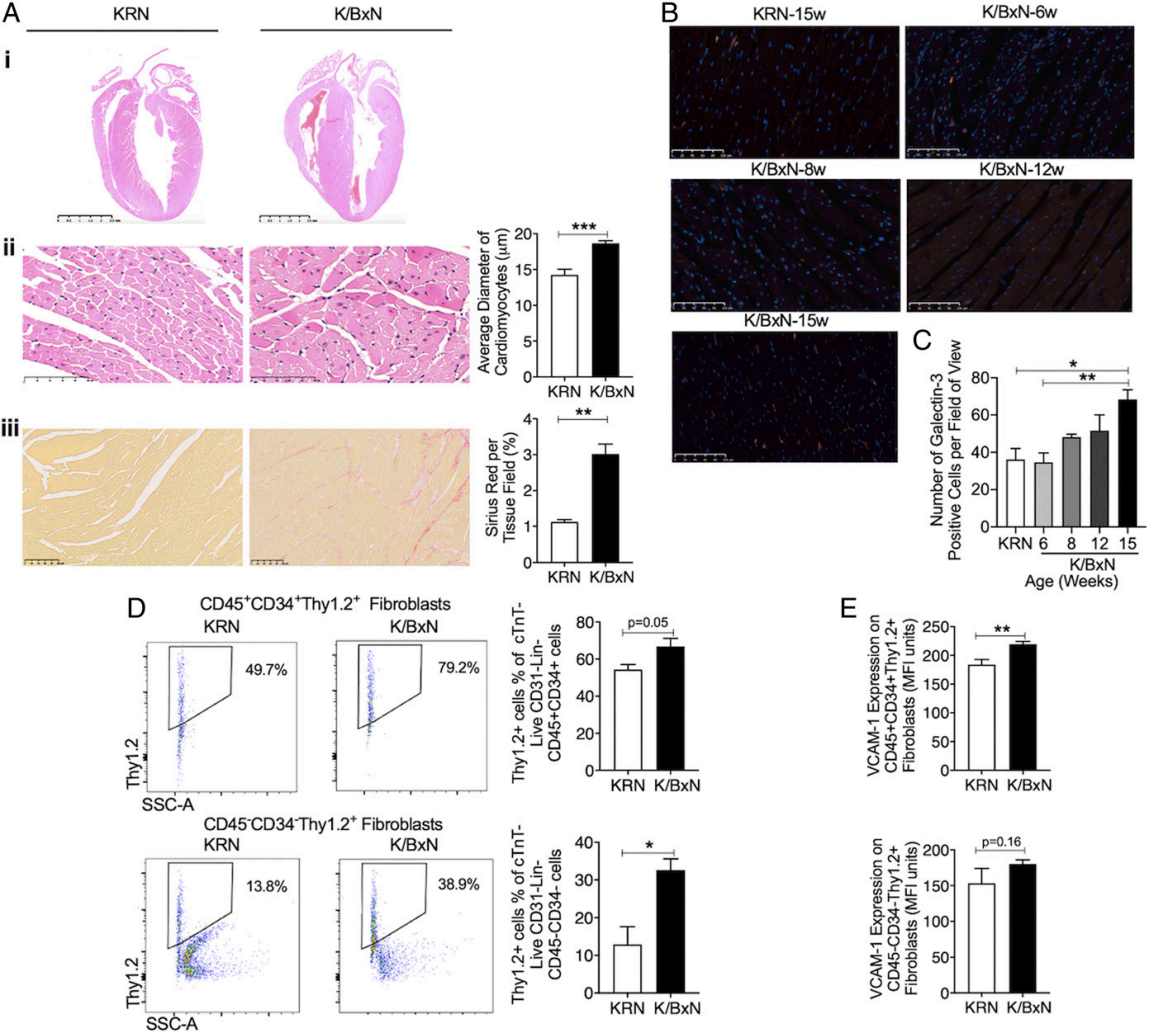
Arthritic K/BxN F1 mice develop cardiac hypertrophy and fibrosis. (*A*) Histological and structural analyses in arthritic hearts (week 15). *A*, i: Macroscopic view of the arthritic mouse heart stained with hematoxylin–eosin. (Scale bars, 2.5 mm.) *A*, ii: Representative LV sections stained with hematoxylin–eosin. *A*, iii: Sirius red from arthritic and nonarthritic hearts. (Scale bars, 100 μm.) KRN: *n* = 3; K/BxN F1: *n* = 6. (*B* and *C*) Quantification of galectin-3–positive cells in LV. Representative images are shown. (Scale bars, 100 μm.) *n* = 3 to 4 per group. (*D*) Quantification of fibroblast populations with representative dot plots and (*E*) VCAM-1 expression for KRN *n* = 4 and K/BxN F1 *n* = 5 at week 8. Data are mean ± SEM (*A*, *D*, and *E*) *P < 0.05, **P < 0.01, ***P < 0.001 versus nonarthritic KRN group (unpaired Student’s *t* test); (*C*) *P < 0.05, **P < 0.01 versus nonarthritic 15-wk-old KRN or 6-wk-old K/BxN F1 group (one-way ANOVA followed by Bonferroni’s test). K/BxN: K/BxN F1.

To gain mechanistic insights into the pathology of the impaired LV relaxation of K/BxN F1 mice, we investigated whether these hearts develop hypertrophy and/or fibrosis, focusing on week 15 at first, when the disease is fully manifested. When compared with age-matched KRN mice, there was a significant increase in interventricular septum thickness in K/BxN F1 mice, but no difference was observed in LV internal diastolic dimensions (Fig. 1*E*), indicating the development of concentric hypertrophy of arthritic hearts. Histological analysis showed that the average diameter of cardiomyocytes was increased in K/BxN F1 hearts compared with KRN hearts (Fig. 2*A*). For fibrosis, we observed higher collagen deposition in K/BxN F1 hearts compared with KRN (Fig. 2*A*). Moreover, galectin-3, which is associated with cardiac fibrosis (21), was significantly increased (Fig. 2 *B* and *C*).

**Fig. 3.**
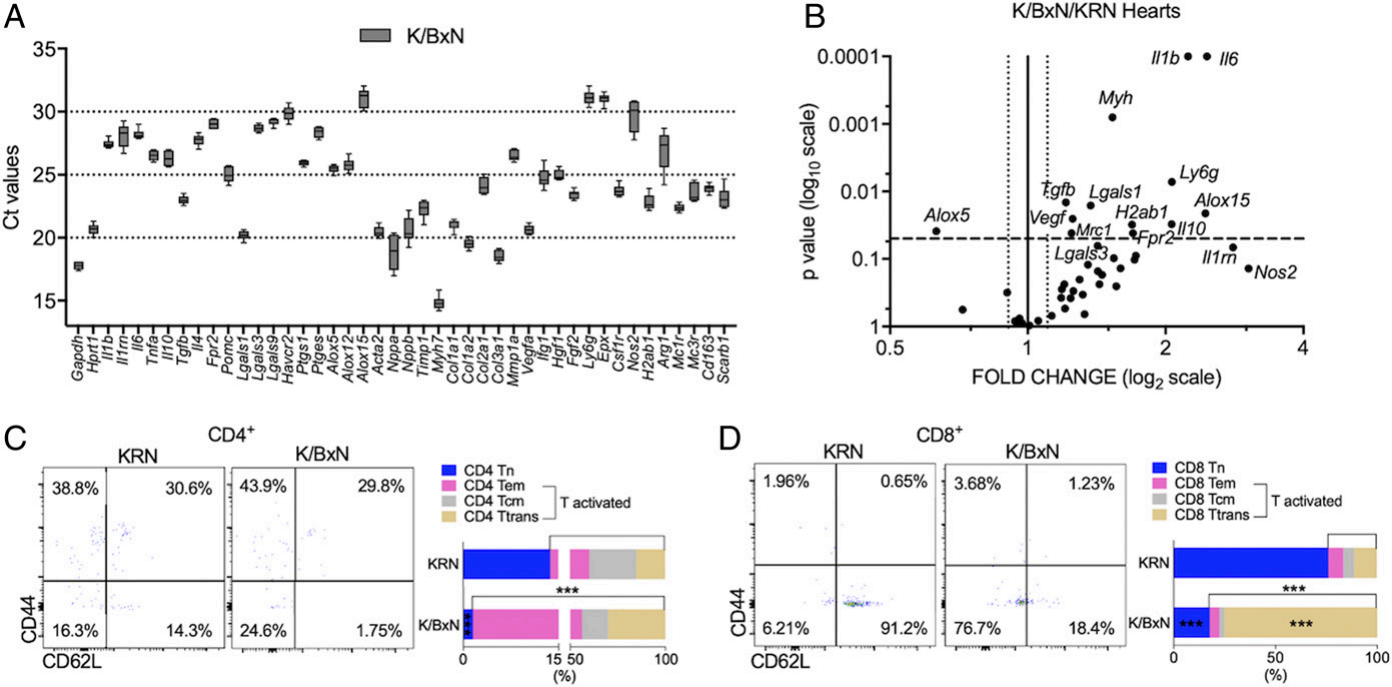
Modulated gene signature and increased infiltration of activated T cells in K/BxN F1 arthritic hearts at 8 wk. (*A*) Ct values of different genes analyzed by qPCR in K/BxN F1 arthritic hearts at week 8. (*B*) Differential gene expression in arthritic hearts at week 8. Ct values were normalized using *Hprt1* as housekeeping gene, and fold change was calculated relative to age-matched KRN controls. *x*-axis shows fold change (in log_2_ scale), with continued line (X = 1) showing KRN mean and dotted lines presenting technical variance: ± average SD of replicated PCR measures. *y*-axis shows the *P* value (in log_10_ scale) from *t* test statistical analysis, with dotted line at *P* = 0.05; KRN group: *n* = 4; K/BxN group: *n* = 6. (*C* and *D*) CD4^+^ (*C*) or CD8^+^ (*D*) T cells were subdivided into non-proinflammatory CD44^−^CD62L^+^ naïve T cells (Tn), CD44^+^CD62L^−^ effector memory T cells (Tem), CD44^+^CD62L^+^ central memory (Tcm), and CD44^−^CD62L^−^ transition status (Ttrans). Data are mean values. KRN group: *n* = 4; K/BxN F1 group: *n* = 5. ***P < 0.001 versus nonarthritic KRN group (unpaired Student’s *t* test). K/BxN: K/BxN F1.

To gain information ahead of established fibrosis, we conducted further analyses at week 8. Both CD45^+^CD34^+^Thy1.2^+^ monocytic-derived fibroblasts (22) and CD45^−^CD34^−^Thy1.2^+^ structural fibroblasts (23) were increased in K/BxN F1 hearts at 8 wk compared with age-matched KRN hearts (*SI Appendix*, Fig. S3A and Fig. 2*D*). VCAM-1, an indicator of proinflammatory status of fibroblasts (21), was significantly increased in monocytic-derived fibroblasts in K/BxN F1 hearts (Fig. 2*E*). Analysis of gene expression profiles in K/BxN F1 hearts confirmed the changes described above with higher messenger ribonucleic acid (mRNA) expression of fibrosis markers including transforming growth factor beta (*Tgfb1*) (Fig. 3 *A* and *B*) as well as cardiac hypertrophy markers including β-myosin heavy chain (*Myh*) and vascular endothelial growth factor (*Vegf*) (Fig. 3 *A* and *B*). No differences in average diameter of cardiomyocytes or collagen deposition were detected at 8 wk between KRN and age-matched K/BxN F1 mice (*SI Appendix*, Fig. S3 *C* and *D*), in line with our echocardiographic findings that full-blown cardiac hypertrophy is marked at week 15 (Fig. 1*E*).

### Inflammatory Markers Are Modulated in Hearts of Arthritic Mice.

When compared with age-matched KRN hearts, K/BxN F1 hearts displayed increased mRNA expression of cytokines including *Il6*, *Il1b*, and *Il10* at week 8 (Fig. 3 *A* and *B*). We also monitored a selection of proresolving receptors, quantifying higher mRNA expression for *Fpr2*. Additionally, the key biosynthetic enzymes of proresolving lipid mediators, 5- and 15-lipoxygenase, were dysregulated (*Alox5* and *Alox15*), while galectin-1 (*Lgals1*) gene product was up-regulated (Fig. 3 *A* and *B*). In the kidney, significant differences were only detected in MHC-II (*H2-ab1*), TIM-3 (*Havcr2*), and *Il1b* mRNA as compared to week 8 nonarthritic KRN mice. These modest changes were unlikely of significance as plasma creatinine levels were not increased even at a later time-point (week 15) in K/BxN F1 mice; rather, they were significantly decreased (*SI Appendix*, Fig. S4), probably due to minimal changes in body weights (Fig. 1*A*).

### Infiltration of Activated T Cells Is Increased in Arthritic Hearts.

Next, we investigated whether cardiac immune cell numbers were altered in these experimental settings (*SI Appendix*, Fig. S3 *B–D*). When compared with age-matched KRN hearts, noninflammatory CD44^−^CD62L^+^ naïve CD4 T cells were decreased in K/BxN F1 hearts at 8 wk (Fig. 3*C*), whereas total numbers of activated CD4 T cells, the sum of CD44^+^CD62L^−^ effector memory, CD44^+^CD62L^+^ central memory, and CD44^−^CD62L^−^ transition status CD4 T cells, were increased (Fig. 3*C*). The same pattern emerged for CD8 T cells (Fig. 3*D*). Week 8 analysis of other immune cell types including cardiac CD115^+^ monocytes, CD11b^+^Ly6G^+^ neutrophils, CD11b^+^SiglecF^+^ eosinophils, F4/80^+^ macrophages, and total CD4^+^ and CD8^+^ T cells in K/BxN F1 hearts revealed no difference from nonarthritic KRN hearts (*SI Appendix*, Fig. S5).

### Myocardial AnxA1 Does Not Increase over Time in Arthritic Hearts.

Immunostaining for AnxA1 in myocardial sections revealed no changes in protein expression over time in the K/BxN F1 hearts (*SI Appendix*, Fig. S6). Intriguingly, the immunostaining for this proresolving mediator increased over time in nonarthritic KRN mice, indicating a time-related change possibly linked with aging, which may be defective in the context of inflammatory arthritis.

### hrAnxA1 Treatment (Week 4 to Week 8) Halted Development of Diastolic Dysfunction in K/BxN F1 mice.

**Fig. 4.**
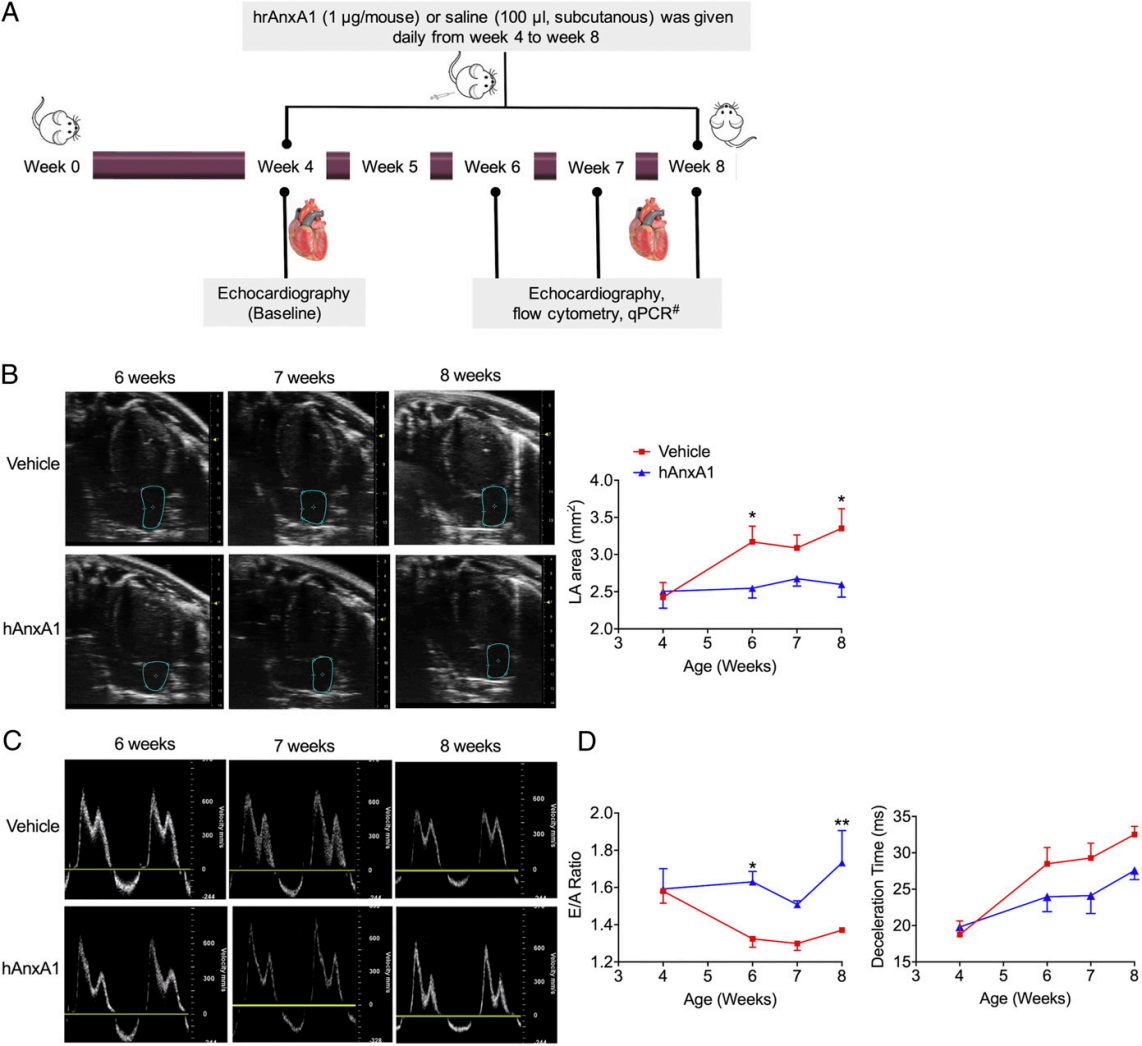
hrAnxA1 treatment (week 4 to week 8) halts the development of diastolic dysfunction and attenuates cardiac hypertrophy in K/BxN F1 mice. (*A*) Schematic indicating treatment and experimental regime. (*B*) Representative B-mode four-chamber echocardiograms indicating LA area and LA atrial area in mice treated with hrAnxA1 or vehicle. (*C*) Representative mitral flow patterns from pulsed-wave color Doppler echocardiography. (*D*) E/A ratio and deceleration time as assessed by echocardiography in arthritic mice treated with hrAnxA1 or vehicle. Data are mean ± SEM. K/BxN F1 + vehicle group: *n* = 5; K/BxN F1+ hrAnxA1 group: *n* = 5. *P < 0.05, **P < 0.01 versus vehicle group (two-way ANOVA followed by Bonferroni’s test). Data are mean ± SEM. K/BxN F1+ vehicle group: *n* = 4; K/BxN F1 + hrAnxA1 group: *n* = 5. *P < 0.05 versus vehicle group (unpaired Student’s *t* test). Veh: vehicle; hAnxA1: hrAnxA1. Echocardiography, flow cytometry, and qPCR were all analyzed blindly.

In the second part of the study, we investigated a proof-of-concept intervention on the pathology unveiled here. Having detected that proresolving targets, particularly FPR2, were up-regulated in the hearts of K/BxN F1 mice, we then tested the potential cardioprotective effects of the FPR2 agonist hrAnxA1 (24). Daily treatment with this protein (1 µg/mouse; Fig. 4*A*) occurred from week 4 (mice with arthritis but no diastolic dysfunction) and terminated at week 8 (mice with both arthritis and diastolic dysfunction).

When compared with K/BxN F1 mice receiving vehicle, hrAnxA1 treatment did not impact the clinical signs of arthritis, such as arthritic scores and body weight loss (*SI Appendix*, Fig. S7 *A* and *B*). Modest yet significant reductions in arthritic scores over baseline values (Δ arthritic score) were observed at only two time-points (day 16 and 18 from start of treatment; *SI Appendix*, Fig. S7*A*). Much more pronounced effects were quantified on the cardiomyopathy associated with this aggressive arthritis.

When compared with the vehicle group, treatment with hrAnxA1 significantly attenuated LA area enlargement and the reduced E/A ratio as early as week 6, that is, 2 wk posttreatment. These beneficial effects lasted until week 8 (Fig. 4 *B–D*), indicating that this pharmacological treatment successfully halted the development of the diastolic dysfunction associated with inflammatory arthritis. No significant alterations in EF, interventricular septum thickness, or LV internal diastolic dimensions were observed in hrAnxA1-treated arthritic mice (*SI Appendix*, Fig. S7 *C* and *D*).

To investigate the potential hemodynamic effects of hrAnxA1, K/BxN F1 mice were treated with hrAnxA1 or vehicle daily from week 7 to week 8. When compared with K/BxN F1 mice receiving vehicle, treatment with hrAnxA1 did not impact mean arterial blood pressure, systolic pressure, diastolic pressure, or heart rate (*SI Appendix*, Fig. S7 *E–H*). Thus, the cardioprotective effects of hrAnxA1 in K/BxN F1 mice are unlikely to be mediated by modulation of blood pressure or heart rate.

### hrAnxA1 (Week 4 to Week 8) Modulates Cellular and Inflammatory Markers in K/BxN F1 Hearts.

**Fig. 5.**
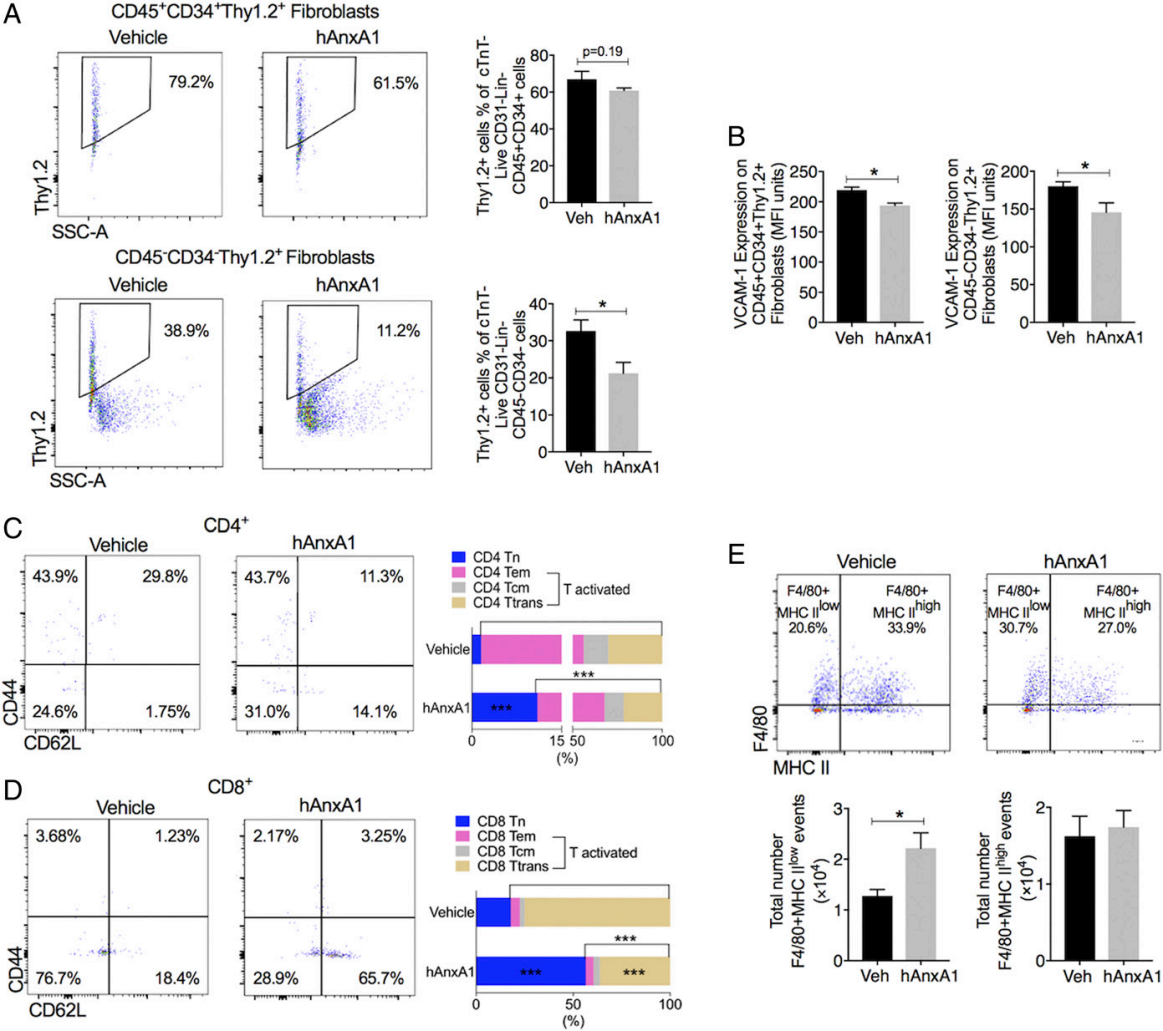
hrAnxA1 treatment (week 4 to week 8) reduces fibroblast populations and activated T cell infiltration and increases MHC II^low^ macrophages in the hearts of K/BxN F1 mice. K/BxN F1 mice were treated with hrAnxA1 [1 µg/mouse daily subcutaneously (s.c.) from week 4 to week 8]. Hearts were enzymatically digested, and cardiac cell phenotype assessed and quantified by flow cytometry. (*A*) Representative dot plots showing cardiac monocytic fibroblasts (CD45^+^CD34^+^Thy1.2^+^) and cardiac structural fibroblasts (CD45^−^CD34^−^Thy1.2^+^) in mice receiving vehicle or hrAnxA1. Cumulative data indicating percentage cardiac monocytic fibroblasts and cardiac structural fibroblasts in mice treated with vehicle or hrAnxA1. (*B*) VCAM-1 expression on cardiac fibroblast subtypes. (*C* and *D*) Representative dot plots and cumulative data illustrating CD4 (*C*) and CD8 (*D*) noninflammatory CD44^−^CD62L^+^ naïve T cells (Tn), CD44^+^CD62L^−^ effector memory T cells (Tem), CD44^+^CD62L^+^ central memory T cells (Tcm), and CD44^−^CD62L^−^ transition status T cells (Ttrans). (*E*) Representative dot plots illustrating F/80^+^MHC II^low^ and F/80^+^MHC II^high^ macrophages K/BxN F1 hearts receiving vehicle or hrAnxA1. Cumulative data indicating total numbers of F/80^+^MHC II^low^ and F/80^+^MHC II^high^ macrophages. *n* = 4 to 5 per group. All data are mean ± SEM *P < 0.05, ***P < 0.001 versus K/BxN F1+ vehicle group (unpaired Student’s *t* test). Veh: vehicle; hAnxA1: hrAnxA1.

Treatment of K/BxN F1 mice with hrAnxA1 impacted the cellular composition of the myocardium. Compared to vehicle, a significant reduction in CD45^−^CD34^−^Thy1.2^+^ structural fibroblast numbers was quantified (Fig. 5*A*), with no major alterations in CD45^+^CD34^+^Thy1.2^+^ monocytic fibroblasts (*P* = 0.19, Fig. 5*A*). The activation status of both fibroblast types was attenuated, as indicated by reduced cell surface expression of VCAM-1 (Fig. 5*B*).

With respect to cardiac immune cells, hrAnxA1 significantly reduced both activated CD4 and CD8 T cell numbers, with concomitant increases in naïve CD4 and CD8 T cells (Fig. 5 *C* and *D*). A modulation of macrophage phenotype was evident, with higher counts for F4/80^+^MHCII^−^ macrophages and no significant change for F4/80^+^MHCII^+^ macrophages (Fig. 5*E*). No changes in the proportion of other immune cell types were detected following 4-wk treatment with hrAnxA1 (*SI Appendix*, Fig. S5).

**Fig. 6.**
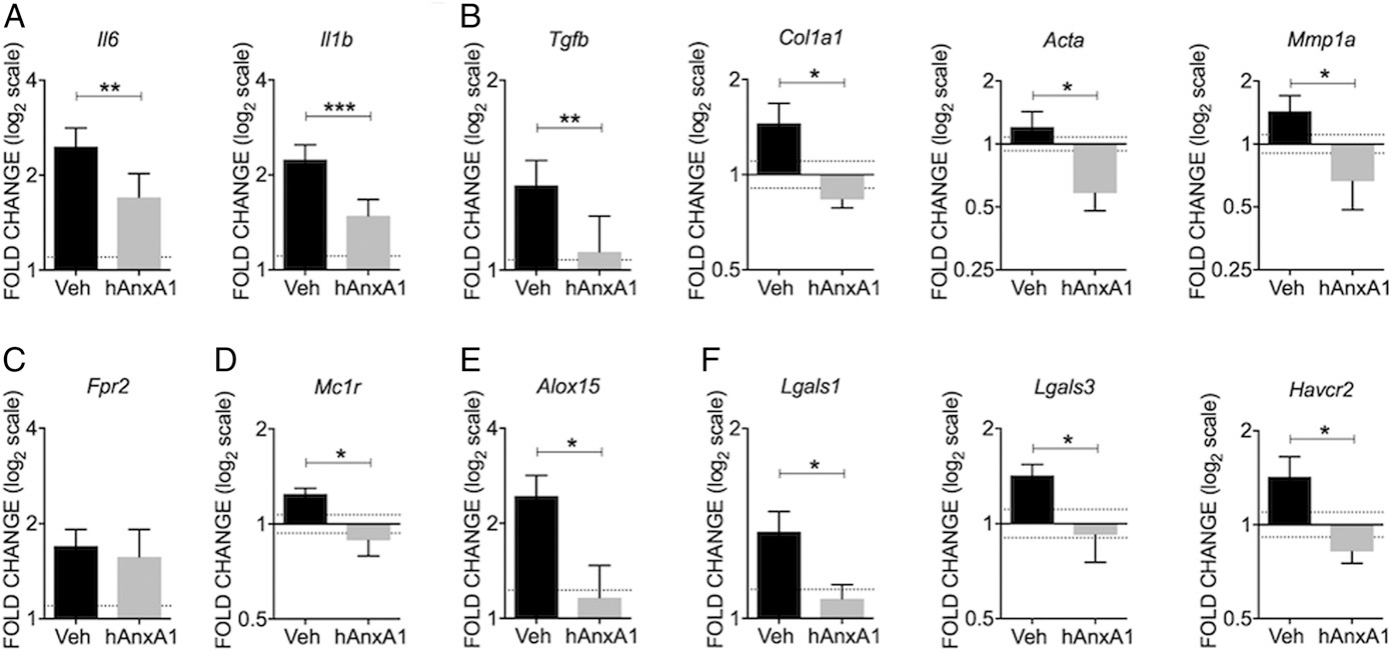
Impact of hrAnxA1 treatment (week 4 to week 8) on cardiac gene expression in K/BxN F1 mice. mRNA expression of (*A*) Proinflammatory cytokines: *Il6* and *Il1b*; (*B*) Cardiac fibrosis markers: *Tgfb*, *Col1a1*, *Acta*, and *Mmp1a*; (*C*) AnxA1 receptor: *Fpr2*; (*D*) Melanocortin receptor 1: *Mc1r*; (*E*) 15-lipoxygenase: *Alox15*; and (*F*) Galectin-1: *Lgals1*; Galectin-3: *Lgals3*; TIM-3 (receptor of Galectin-9): *Havcr2*. Ct values were normalized using *Hprt1* as housekeeping gene. Data are mean ± SEM. KRN group: *n* = 4; K/BxN F1 + vehicle group: *n* = 6; K/BxN F1 + hrAnxA1 group: *n* = 6. Fold change was calculated relative to age-matched nonarthritic KRN controls (mean value = 1), with dotted line showing technical variance indicated by ± average SD of replicated PCR measures for each gene. *P < 0.05, **P < 0.01, ***P < 0.001 versus vehicle group (unpaired Student’s *t* test).

In terms of mediator expression, hrAnxA1 significantly reduced cardiac mRNA of proinflammatory cytokines like *Il6* and *Il1b* (Fig. 6*A*). Similarly, significant reductions were quantified for markers of fibrosis including *Tgfb1*, *col1a1* (type 1 collagen), *Acta2* (α-smooth muscle actin), and *Mmp1a* (matrix metalloprotease-1a) (Fig. 6*B*). Such a “normalization” of local inflammation was also evident in relation to the expression of proresolving receptors, mediators and enzymes (Fig. 6 *D–F*). Of interest, *Fpr2* mRNA expression in the heart was not altered by hrAnxA1 treatment (Fig. 6*C*). Congruently, treatment of mice with hrAnxA1 did not alter surface FPR2 expression in blood Ly6G^+^ neutrophils, Ly6C^+^ monocytes, or CD3^+^ lymphocytes in K/BxN F1 mice (*SI Appendix*, Fig. S8). hAnxA1 treatment did not alter mRNA expression of several markers quantified in K/BxN F1 kidneys (*SI Appendix*, Fig. S4).

### hrAnxA1 (Week 8 to Week 15) Reversed Established Diastolic Dysfunction and Attenuated Cardiac Remodeling.

Next, we applied a therapeutic protocol with daily hrAnxA1 treatment (1 µg/mouse) from week 8 (mice with both arthritis and established diastolic dysfunction) to week 15 (mice with arthritis, diastolic dysfunction, and cardiac hypertrophy and fibrosis).

**Fig. 7.**
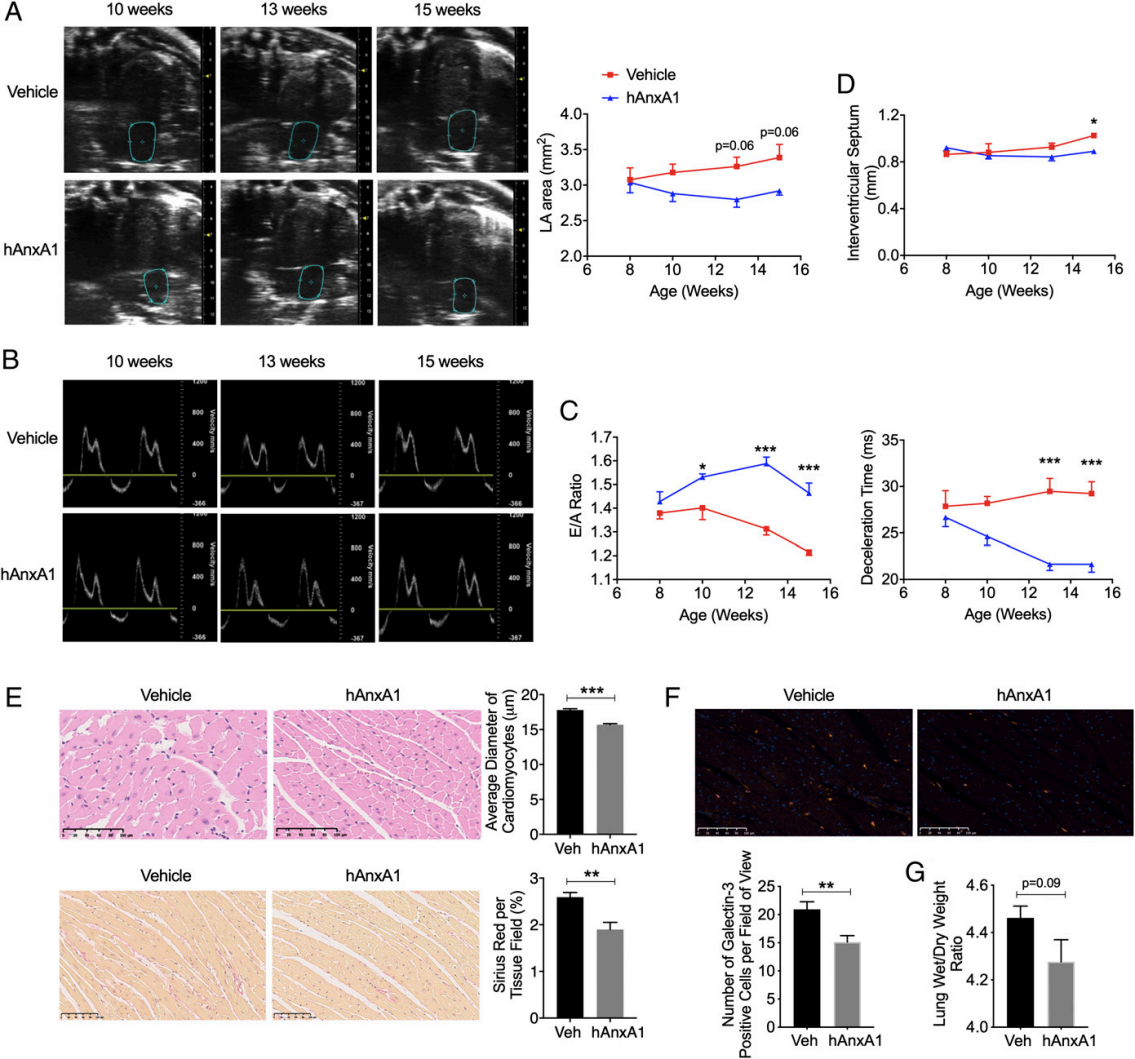
hrAnxA1 treatment (week 8 to week 15) reverses the established diastolic dysfunction and attenuates cardiac remodeling in K/BxN F1 mice. K/BxN F1 mice were treated with either hrAnxA1 (1 μg/mouse, daily subcutaneous injection from week 8 to week 15) or vehicle (100 μL saline). Echocardiography was performed in K/BxN F1 at 8 wk (baseline) before hrAnxA1 or saline administration and at 10, 13, and 15 wk. (*A*) Representative B-mode four-chamber echocardiograms indicating LA area and cumulative data of LA area in mice treated with hrAnxA1 or vehicle. (*B*) Representative mitral flow patterns from pulsed-wave color Doppler echocardiography. (*C*) E/A ratio and deceleration time as assessed by echocardiography in arthritic mice treated with hrAnxA1 or vehicle. (*D*) hrAnxA1 reduced interventricular septum thickness in arthritic mice at 15 wk. Data are mean ± SEM. K/BxN F1 + vehicle group: *n* = 6; K/BxN F1+ hrAnxA1 group: *n* = 6. *P < 0.05, ***P < 0.001 versus vehicle group (two-way ANOVA followed by Bonferroni’s test). (*E*) Representative LV sections stained with hematoxylin–eosin and Sirius red from arthritic hearts received hrAnxA1 or vehicle and respective cumulative data. (Scale bars, 100 μm.) (*F*) Quantification of galectin-3–positive cells in LV. Representative images are also shown. (Scale bars, 100 μm.) (*G*) Lung wet-to-dry weight ratio. *P < 0.05, **P < 0.01, and ***P < 0.001 versus vehicle group (unpaired Student’s *t* test). Veh: vehicle; hAnxA1: hrAnxA1.

When compared with the vehicle group, there was a trend for LA area reduction by hrAnxA1 treatment (*P* = 0.06, Fig. 7*A*). More strikingly, the E/A ratio was significantly increased by hrAnxA1 as early as week 10, that is, 2 wk posttreatment initiation. Deceleration time was significantly shortened by hrAnxA1 from week 13, as compared with vehicle control. These beneficial effects lasted until week 15 (Fig. 7 *B* and *C*). This treatment at a later stage of the experimental disease also impacted cardiac remodeling. Week-15 echocardiography analysis showed that interventricular septum thickness was significantly reduced in the hrAnxA1 group, compared with vehicle (Fig. 7*D*). This was in line with histological analyses which demonstrated a decrease in the average diameter of cardiomyocytes (Fig. 7*E*). Additionally, when compared with vehicle, hrAnxA1 significantly decreased collagen deposition (Fig. 7*E*) and the number of galectin-3–positive cells (Fig. 7*F*) in K/BxN F1 hearts indicative of a negative impact on disease evolution to more advanced cardiac remodeling, including cardiac hypertrophy and fibrosis. A trend, although not significant, for reduction in lung wet to dry weight ratio was calculated for hrAxA1-treated mice (*P* = 0.09, Fig. 7*G*).

**Fig. 8.**
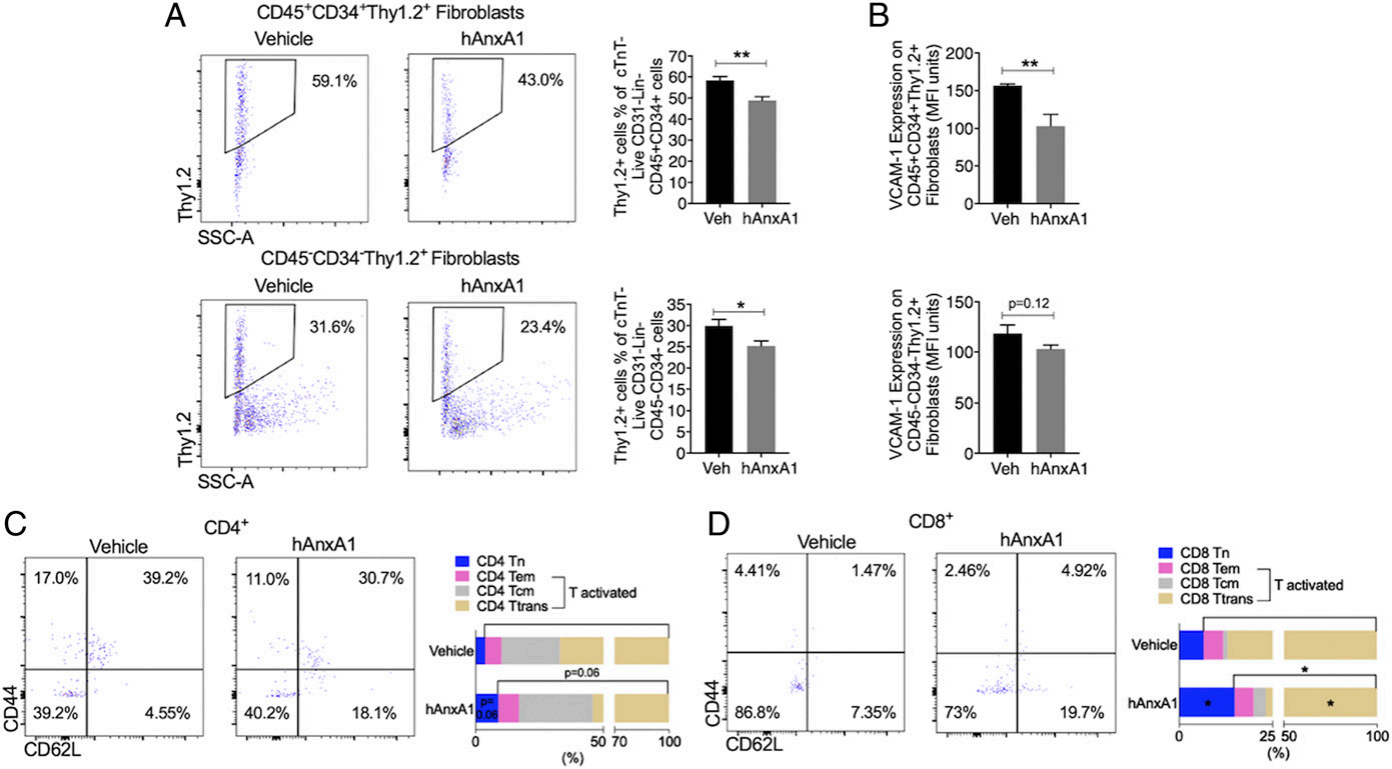
hrAnxA1 treatment (week 8 to week 15) reduces fibroblast populations and activated T cell infiltration in the hearts of K/BxN F1 mice. K/BxN F1 mice were treated with either hrAnxA1 (1 μg/mouse, daily subcutaneous injection from week 8 to week 15) or vehicle (100 μL saline). Hearts were enzymatically digested, and cardiac cell phenotype assessed and quantified by flow cytometry. (*A*) Representative dot plots showing cardiac monocytic fibroblasts (CD45^+^CD34^+^Thy1.2^+^) and cardiac structural fibroblasts (CD45^−^CD34^−^Thy1.2^+^) in mice receiving vehicle or hrAnxA1. Cumulative data indicating percentage cardiac monocytic fibroblasts and cardiac structural fibroblasts in mice treated with vehicle or hrAnxA1. (*B*) VCAM-1 expression on cardiac fibroblast subtypes. (*C* and *D*) Representative dot plots and cumulative data illustrating CD4 (*C*) and CD8 (*D*) noninflammatory CD44^−^CD62L^+^ naïve T cells (Tn), CD44^+^CD62L^−^ effector memory T cells (Tem), CD44^+^CD62L^+^ central memory T cells (Tcm), and CD44^−^CD62L^−^ transition status T cells (Ttrans). *n* = 6 per group. All data are mean ± SEM. *P < 0.05, **P < 0.01 versus K/BxN F1+ vehicle group (unpaired Student’s *t* test). Veh: vehicle; hAnxA1: hrAnxA1.

Treatment of K/BxN F1 mice with hrAnxA1 at a later stage modulated both cardiac fibroblast composition and cardiac immune cells. When compared to vehicle, both CD45^+^CD34^+^Thy1.2^+^ monocytic fibroblasts and CD45^−^CD34^−^Thy1.2^+^ structural fibroblasts were reduced by hrAnxA1 (Fig. 8*A*). The activation marker of fibroblasts, VCAM-1, was reduced in monocytic fibroblasts (Fig. 8*B*), with no major alterations in structural fibroblasts (*P* = 0.12, Fig. 8*B*). When compared with the vehicle group, there was a trend for reduction in activated CD4 T cells, while the naïve CD4 T cells were increased by hrAnxA1 treatment (*P* = 0.06, Fig. 8*C*). hAnxA1 significantly reduced activated CD8 T and increased naïve CD8 T cell numbers in K/BxN F1 hearts (Fig. 8*D*).

Altogether these data indicate that hrAnxA1 treatment successfully reverses the established diastolic dysfunction and associated cellular changes within the hearts that develop with inflammatory arthritis.

## Discussion

In this study, we characterize a model of HFpEF and concentric hypertrophy in severe inflammatory arthritis that manifests after joint disease peaks. Analyses of the hearts of arthritic mice revealed immune as well as structural insufficiency. A partial dissociation of the myocardial phenotype from the joint disease emerges also from the pharmacological studies with hrAnxA1 with modest effects on the arthritic joint but higher efficacy in correcting the diastolic dysfunction. We propose that the K/BxN F1 mouse model can be exploited to identify pathways and therapeutic strategies for the management of the cardiomyopathy that affects RA patients.

In RA patients, diastolic dysfunction is strongly associated with disease duration (25). In our model, diastolic dysfunction was established at week 8, 4 wk later than the fully developed arthritis; this may reflect a chronic myocardial process resulting in impairment of cardiac function. At week 8, the development of diastolic dysfunction in K/BxN F1 mice was confirmed by an enlarged LA area (suggestive of reduced LV compliance), reduced E/A ratio, reduced E wave, and increased deceleration time; these dynamic changes are essentially the same as reported in RA patients (9). The normal renal function detected in K/BxN F1 mice indicates that the cardiac dysfunction was not secondary to cardiorenal syndrome. Additionally, no differences in blood pressure were observed between K/BxN F1 arthritic mice and KRN control mice at week10, 15, or vehicle-treated mice at 8 wk. This outcome is in line with previous studies in rodents with collagen-induced arthritis which showed that blood pressure values were not altered by the disease (26, 27). Increased blood pressure is a common comorbidity in HFpEF patients and is a contributory pathogenic factor in some patients with diastolic dysfunction (28, 29). Furthermore, clinical evidence has shown that the prevalence of hypertension increases with age and disease duration in patients with RA (30). It is therefore likely that hypertension is a comorbidity that contributes to development of diastolic dysfunction in some RA patients. However, in this murine model of diastolic dysfunction in K/BxN F1 mice, our data points to systemic inflammation rather than modulation of blood pressure as the major driving force for the observed cardiac phenotype. While we have not ruled out hypertension in K/BxN F1 mice younger than 8 wk of age, we speculate that it is unlikely that these mice would be hypertensive at weeks 4 through 6 then become normotensive at week 8 onwards after developing diastolic dysfunction. We propose that alterations in systemic blood pressure are not causative of diastolic dysfunction in arthritic mice, at least in the conditions investigated here.

The clinical impact of HFpEF in RA has prompted some investigation into cardiac function in murine models of arthritis. In a recent study (31), cardiac dysfunction in collagen antibody-induced arthritis was reported; these mice developed a systolic alteration, not a diastolic one as reported here and, relevantly, characterized in RA patients. Nonetheless, this important study showed mice with collagen antibody-induced arthritis developed cardiac hypertrophy and fibrosis and isolated cardiomyocytes from these mice displayed impaired Ca^2+^ signaling. In a separate study (32), Zhou et al. reported susceptibility to cardiac systolic dysfunction and dilated cardiomyopathy in mice undergoing an experimental arthritis induced with collagen antibody. Finally, we note how the K/BxN F1 progeny have been used in a single study to investigate secondary organ injury: these authors observed the spontaneous development of endocarditis by week 8. Interestingly, while the arthritic phenotype of K/BxN mice is dependent on complement C5, such a pathway was not required for the development of endocarditis, which relied on FcγR, the absence of which had little impact on arthritis severity (20).

In non-RA settings, the pathology of HFpEF is linked to immune dysregulation and systemic inflammation (28). This results in cardiac remodeling leading to functional changes characterized by LV hypertrophy, fibrosis, and diastolic dysfunction (28). In RA patients, the cardiomyopathy correlates with LV concentric hypertrophy characterized by LV wall thickening without dilation, contributing to diastolic dysfunction and HFpEF (33). These features could be demonstrated in K/BxN F1 mice, which at week 15 develop concentric hypertrophy of the heart (20). Such a macroscopic and significant sign of pathology may plausibly result from molecular changes that occur as early as week 8, as indicated by up-regulated mRNA expression of cardiac hypertrophy markers like β-myosin heavy chain and VEGF.

In RA patients, myocardial fibrosis and inflammation detected by cardiac magnetic resonance imaging are evident even in a proportion of patients with no overt cardiovascular diseases (34), and it is not surprising that the detected cardiac fibrosis and inflammation are associated with the development of CHF (35). In our study, we have quantified an up-regulation of cardiac proinflammatory cytokines in K/BxN F1 mice; this could be the result of a 1) direct impact of systemic inflammation (e.g., higher circulating cytokines) (28) and/or 2) local inflammation provoked by interaction of glucose-6-phosphate isomerase autoantibodies activating Fc receptors (20). In parallel, collagen accumulation was increased in these hearts indicating presence of fibrosis, possibly the end-point of 1) elevated numbers of both cardiac monocytic and structural fibroblasts and 2) their increased activity indicated by elevated *Tgfb1* mRNA and VCAM-1 protein expression. In line with our findings, increased monocytic fibroblasts derived from the bone marrow can mediate cardiac fibrosis in a model of angiotensin II-induced cardiac hypertrophy (22). Up-regulation of adhesion molecules on cardiac fibroblasts plays a key role in recruitment and infiltration of immune cells which contributes to heart inflammatory disorders including CHF (36). The increased surface expression of VCAM-1 in cardiac fibroblasts might be secondary to up-regulated cardiac proinflammatory cytokines (36) in the arthritic mice. Increased VCAM-1, in turn, may have promoted recruitment of activated T cells through VCAM-1/α4β1 integrin interaction (37), forming a feed-forward mechanism and amplifying the inflammatory reaction within arthritic hearts. The link between T cells and diastolic dysfunction or HFpEF has not been fully elucidated. We report here an increased proportion of activated T cells associated with and potentially contributory to the development of diastolic dysfunction. Other studies suggest that activated T cells play a pivotal role in nonischemic, pressure overload–induced cardiomyopathy as well as in rejection of heart allografts, as activated T cell infiltration into the LV predisposes LV inflammation, hypertrophy, fibrosis, and systolic dysfunction; this is likely to be of functional relevance because T cell inhibition or deficiency alleviated these pathological changes (38–40).

Having characterized at least some elements of this model of HFpEF in KBxN F1 mice, the second part of the study was directed to use it as proof-of-concept for therapeutic investigation. Since persistent inflammation leading to fibrosis may be due not only to excessive proinflammatory mediators but also to defects in endogenous proresolving mechanisms (11), we monitored specific effectors of resolution including galectin-1, FPR2, and 5- and 15-lipoxygenase (11). We focused on FPR2–up-regulated in the arthritic mice, because this receptor is emerging as a target for cardio protection with an on-going Phase I trial for a small molecule agonist (ClinicalTrials.gov Identifier: NCT03335553) (41). Moreover, an FPR1/FPR2 dual agonist, compound 17b, preserves cardiac function in models of acute myocardial infarction (42), while another dual agonist, compound 43, affords cardio protection in experimental heart failure induced by permanent coronary artery ligation through its ability to modify macrophage phenotype (43). We have a long-dated interest in FPRs and their agonist AnxA1; the heart can be targeted by AnxA1 and AnxA1 peptide Ac2-26 protecting against myocardial ischemia reperfusion injury (15, 44). These cardio-protective effects are mediated by FPR2 as demonstrated by loss of efficacy after administration of receptor antagonists (44) and persistence of efficacy in FPR1 null mice (45). More recently, hrAnxA1 was showed to protect against myocardial inflammation in type I diabetes–induced cardiomyopathy, a result obtained through dampening cardiac MAPK signaling and activating the prosurvival Akt pathway (24).

Based on this background and due to the translational potential inherent of this receptor target, we tested the effect of daily administration of hrAnxA1 in arthritic K/BxN F1 mice observing a remarkable attenuation of the diastolic cardiomyopathy. It is difficult to tease out the site of action; the fact that the aggressive arthritis of the joint was only minimally affected by the treatment suggests that cardio protection may occur independently from any amelioration of joint disease. Rather, it could derive from effects downstream of changes in the circulation or the heart itself.

The role of AnxA1 in experimental arthritis has been reported with different outcomes depending on the model and the route of administration. In the collagen-induced arthritis model, administration of hrAnxA1 during the immune phase augmented the severity of arthritis in the effector phase, probably through a positive action on the T cell response (46). Contrasting data have been reported though (47), and this discrepancy has been discussed (48) but to our knowledge not solved. It may lie on the fine regulation of the interaction between dendritic cells and T cells. Our experience with the serum-transfer model of arthritis did not highlight powerful inhibitory properties of hrAnxA1 on joint inflammation, but 1) a cleavage-resistant AnxA1 was effective in attenuating joint disease, and 2) dexamethasone lost most of its efficacy in AnxA1 null mice (49). In summary, the pharmacological properties of AnxA1 vary across models and experimental protocols. In the model of K/BxN F1 arthritis used here, we reason that the high severity of the joint disease precluded the possibility to unveil direct anti-arthritic effects of hrAnxA1.

A different scenario emerged at the level of the heart. Treatment of mice with hrAnxA1 drastically reduced proinflammatory cytokine transcripts including IL-6 and IL-1β. Moreover, the treatment impacted on cellular events selectively. Myocardial inflammation and fibrosis are associated with the development of CHF in RA patients (35). In these experiments, hrAnxA1 reduced fibroblast populations and profibrotic markers. The reduced fibroblast surface expression of VCAM-1 could be the consequence of both an indirect effect of hrAnxA1 through reduced inflammation (36) and a direct action on the cell. FPR2 is expressed in cardiac fibroblasts (50) as well as lung and renal fibroblasts (51, 52). In lung fibroblasts, AnxA1 mimetics regulate TNF-induced proliferation and inflammatory responses in an FPR2-dependent manner (51). Additionally, overexpression of AnxA1 attenuates profibrotic activity of TGF-β-treated renal fibroblasts (52). In our settings, the reduced myocardial inflammation may decrease surface expression of VCAM-1 in cardiac fibroblasts (36), which in turn reduces activated T cell recruitment (37), promoting in this manner a therapeutic feed-forward circuit that further dampens myocardial inflammation upon AnxA1 treatment in K/BxN F1 mice. Additionally, FPR activation by compound 43 inhibits the expansion of activated CD4 T cells in murine models of inflammatory arthritis (53), which at least in part might explain the reduced infiltration of activated T cells in K/BxN F1 hearts after treatment with hrAnxA1.

Moreover, hrAnxA1 increased cardiac MHC II^low^ macrophages in K/BxN hearts. FPR2 is highly expressed in macrophages (12); therefore, the shift toward MHC II^low^ macrophages upon hrAnxA1 treatment may be due to direct action on this cell type. In settings of muscle injury, hrAnxA1 favors tissue repair by controlling macrophage phenotype switch, an effect downstream of FPR2 activation and associated with AMPK activation (54). The same study revealed how human monocyte-derived macrophages express high levels of FPR2 when polarized to an inflammatory phenotype, with much lower expression of the receptor protein and gene transcript, when polarized toward a reparative macrophage. In other words, FPR2 can act as a switch for macrophage reactivity and sensor of the tissue environment. Within cardiac tissue, macrophage subsets are defined on expression of MHC II and CCR2 supported by both transcriptional and functional analysis as well as a single-cell RNA-sequencing study (55, 56). MHC II^low^ macrophages display higher protease and matrix metalloprotease activity which enhances matrix breakdown, whereas MHC II^high^ macrophages are enriched for a gene set that regulates profibrotic TGF-β and promote fibrosis (57). In line with our findings, increased MHC II^low^ macrophages are associated with improved diastolic dysfunction induced by hypertension in mice (57). Of relevance, in settings of myocardial infarction, hrAnxA1 afforded cardio protection by promoting local angiogenesis through polarization of a specific macrophage subtype (58). This seems to be different from what is observed here, though more focused analyses may be required. Therefore, as for the fibroblasts and irrespective of whether a direct or indirect effect of hrAnxA1 regulates macrophage phenotype, these cellular events and the fine-tuning of their activation status can chiefly contribute to the protection afforded by this mediator against the diastolic cardiomyopathy.

Treatment with hrAnxA1 from week 8 to week 15 not only successfully reversed the established diastolic dysfunction associated with inflammatory arthritis but also attenuated cardiac hypertrophy and fibrosis. These protective effects were, at least in part, contributed by the reduction in count and the activation status of cardiac fibroblasts and cardiac immune cells.

It is noteworthy that prolonged treatment with hrAnxA1 did not affect expression of FPR2 on circulating white blood cells. This result is aligned with the lack of reduction in heart *Fpr2* mRNA expression even when the diastolic dysfunction was significantly corrected (suggesting that elevated cardiac F*pr2* mRNA was not contributing to the heart inflammation and fibrosis but possibly part of a frustrated circuit aiming to dampen these pathological events). Altogether, these observations may have bearing on the development of AnxA1 mimetics or FPR2 small-molecule agonists, suggesting that repeated treatment with the proper frequency and dosage should not lead to receptor down-regulation and desensitization. Clearly, this needs to be substantiated by future experiments.

The complex nature of inflammatory arthritis and its secondary injury to other organs (the heart under focus here, but also, lungs and kidneys can be affected in chronic settings) is more complex than simply due to a “sustained inflammatory phase.” We propose that a modulation of the host inflammatory response rather than inhibiting inflammation through classic anti-inflammatories could yield better therapeutic outcomes. Instead of merely inhibiting inflammation, resolution of inflammation is characterized in multiple effects on immune and stromal cells through downstream positive/activating mechanisms (11). In the settings of nonresolving chronic inflammatory arthritis presented here, hrAnxA1 not only reduced proinflammatory cytokines and profibrotic markers in the heart but also decreased activated cardiac T cells and induced a macrophage–phenotype switch which may explain why this therapeutic approach can have an advantage over classic anti-inflammatories.

In conclusion, we have characterized a mouse model of inflammatory arthritis, K/BxN F1 progeny, in which the specific diastolic dysfunction with preserved EF typical of RA patients is recapitulated. This diastolic dysfunction is associated with cardiac hypertrophy and myocardial inflammation and fibrosis. Maintained expression of a specific proresolving receptor in the diseased hearts prompted us to test administration of hrAnxA1. Indeed, hrAnxA1 halted the progression of cardiac diastolic dysfunction in KBxN F1 mice without substantial modulation of the clinical severity of arthritis. Therefore, we envisage a cotherapy approach to halt aggressive arthritis and prevent cardiomyopathy. The cardio protection exerted by hrAnxA1 in these mice is associated with 1) reduced cardiac fibroblast populations and profibrotic markers; 2) modulated cardiac immune cells including decreased activated T cell infiltration and increased MHC II^low^ macrophages; and 3) down-regulated proinflammatory cytokines. Thus, we propose that hrAnxA1 and/or FPR2 agonists, as prototypical exemplars of resolution pharmacology, may offer an approach to the treatment of diastolic dysfunction or HFpEF associated with inflammatory arthritis.

## Materials and Methods

Additional details on the methods are provided in *SI Appendix*.

### Animals.

All animal procedures were performed in accordance with the institutional Animal Welfare Ethical Review Body and UK Home Office guidelines. Details of the colonies used and animal welfare are available in *SI Appendix*.

### Assessment of LV Diastolic and Systolic Function In Vivo.

M-mode and Doppler echocardiography were performed using a Vevo-3100 imaging system to analyze systolic and diastolic function, respectively. Cardiac function in hrAnxA1 treatment studies was assessed blindly. Details are available in *SI Appendix*.

### Assessment of Arthritic Scores.

Disease was monitored by assessing the clinical score as described previously (14). Arthritic scores in hrAnxA1 treatment studies were assessed blindly. Details are available in *SI Appendix*.

### Quantification of Cardiomyocyte Size and Collagen Deposition.

Diameters of cardiomyocytes were measured using hematoxylin–eosin sections. Collagen deposition was determined using Sirius red-stained sections as detailed in *SI Appendix*. Cardiomyocyte size and collagen deposition in hrAnxA1 treatment study were assessed blindly.

### Flow Cytometry.

Flow cytometry of heart tissue was performed as detailed in *SI Appendix*. All flow cytometry analyses were carried out blindly.

### RT-PCR RNA Quantification.

The mRNA expression of exemplar proinflammatory, proresolving, fibrosis, and hypertrophy markers were determined in whole mouse heart or kidney tissue. All RT-PCR analyses were conducted blindly. Details are available in *SI Appendix*.

### Statistics.

All values described in the text and figures are presented as mean ± SEM or mean values of n observations. One-way ANOVA followed by Bonferroni’s post hoc test or unpaired Student’s *t* test were used to compare intergroup differences. Two-way ANOVA followed by Bonferroni’s post hoc test was used to compare time-courses. In all cases, a *P* < 0.05 was considered statistically significant.

## Supplementary Material

Supplementary File

Supplementary File

Supplementary File

Supplementary File

Supplementary File

Supplementary File

Supplementary File

Supplementary File

## Data Availability

All study data are included in the article and/or supporting information.
