# Supplementary material for: Annexin A1 attenuates cardiac diastolic dysfunction in mice with inflammatory arthritis
Source: Proc Natl Acad Sci U S A. 2021 Sep 15;118(38):e2020385118. doi: 10.1073/pnas.2020385118 (PMC8463875; doi:10.1073/pnas.2020385118)
Supplement: Supplementary File [file pnas.2020385118.sapp.pdf]

Supplementary Material

Annexin A1 attenuates cardiac diastolic dysfunction in mice with inflammatory arthritis

Jianmin Chen MD, PhD[1#], Lucy V Norling PhD[1#], Jose Garrido Mesa PhD[1], Marina De Paula Silva PhD[1], Sophie E Burton[1], Chris Reutelingsperger PhD[2], Mauro Perretti PhD[1†], Dianne Cooper PhD[1†]

[1] William Harvey Research Institute, Bart's and The London School of Medicine, Queen Mary University of London, London, UK
[2] Department of Biochemistry, Cardiovascular Research Institute, Maastricht University, Maastricht, The Netherlands, 6229HX

[#] These authors share first authorship.
[†] These authors share senior authorship.

MATERIALS AND METHODS

Animals

All animal procedures were performed in accordance with the institutional Animal Welfare Ethical Review Body and UK Home Office guidelines. KRN T cell receptor transgenic mice (gift from Mohini Gray, Edinburgh University, Edinburgh, United Kingdom) were maintained on a C57BL/6 background (K/B). Arthritic mice (K/BxN F1) were obtained by crossing homozygous K/B with NOD/ShiLtJ mice (N) (Charles River) and they express both the T cell receptor transgene KRN and the major histocompatibility complex (MHC) class II molecule A^{g7} from the NOD/ShiLtJ mouse strain. Age-matched KRN T cell receptor transgenic mice on a C57BL/6 background were used as controls in all experiments. All mice were bred in house and maintained on a standard chow pellet diet and had access to water ad libitum, with a 12-hour light-dark cycle. Cages had clean bedding and cardboard rolls to allow dark areas for mice to shelter.

Power calculations for number of animals required were conducted using the experimental design assistant (EDA) (https://eda.nc3rs.org.uk). Detection of diastolic dysfunction measured by left atrial area changes in mice with inflammatory arthritis indicated that 5 to 6 mice are sufficient to expose a 1 mm^2 increase in left atrial area. In total 87 K/BxN F1 mice and 47 KRN mice were used for the study. Mice were excluded from the study if they lost 20% of their body weight from start of the experimental period (2 KBxN F1 were excluded). For each animal treatment administration was performed by an investigator not involved in the data analysis process and was the only researcher aware of treatment group allocation. A different investigator was responsible for outcome assessment and data analysis and was not involved in treatment administration. To conduct a time-course study investigating onset and persistence of the cardiac dysfunction in K/BxN F1 mice, the following two groups were studied: (i) KRN (n=5); (ii) K/BxN F1 (n=6). In the hrAnxA1 therapeutic study from week 4 to week 8, K/BxN F1 mice were randomly allocated into two different groups for echocardiography and flow cytometry in the heart: (i) K/BxN F1 + vehicle (n=5); (ii) K/BxN F1 + hrAnxA1 (1 µg/mouse, daily subcutaneous injection from week 4 to week 8, n=5) (1). Four age-matched KRN mice were used as control for flow cytometry. In a separate hrAnxA1 treatment experiment from week 4 to week 8, K/BxN F1 mice were randomly allocated into two different groups for real-time PCR (RT-PCR) RNA quantification in the heart with hrAnxA1 treatment: (i) K/BxN F1 + vehicle (n=6); (ii) K/BxN F1 + hrAnxA1 (same treatment protocol as above, n=6). Four age-

matched KRN mice were used as control. In the hrAnxA1 therapeutic study from week 8 to week 15, K/BxN F1 mice were randomly allocated into two different groups for echocardiography and flow cytometry in the heart: (i) K/BxN F1 + vehicle (n=6); (ii) K/BxN F1 + hrAnxA1 (1 μg/mouse, daily subcutaneous injection from week 8 to week 15, n=6). Vehicle and hrAnxA1 treated mice were housed together (max 5 mice per cage) and selected at random each day for clinical assessment of arthritis and treatment to minimise confounders.

Assessment of left ventricular diastolic and systolic function *in vivo*

M-mode and Doppler echocardiography were performed in K/BxN F1 mice and age-matched KRN mice at 4, 6, 8, 10, 12 and 15 weeks for time-course cardiac function analysis. In hrAnxA1 therapeutic study from week 4 to week 8, echocardiography was performed in K/BxN F1 at 4 weeks (baseline) before hAnxA1 or saline administration, and at 6, 7 and 8 weeks. In the hrAnxA1 therapeutic study from week 8 to week 15, echocardiography was performed in K/BxN F1 at 8 weeks (baseline) before hAnxA1 or saline administration, and at 10, 13 and 15 weeks. Anesthesia was induced with 3% isoflurane and maintained at 0.5 to 0.7 % for the duration of the procedure. Before assessment of cardiac function, fur was removed from the chest area to allow accurate assessment of cardiac function and mice were allowed to stabilize for at least 10 minutes. Body temperature was maintained at 37 °C. During echocardiography, the heart rate was measured from electrocardiogram and were kept consistent between experimental groups (400-500 bpm). Echocardiography images were recorded using a Vevo-3100 imaging system with a 40-MHz linear probe (VisualSonics, Toronto, Canada). Diastolic transmitral left ventricle (LV) inflow images were acquired from apical four-chamber views using pulsed-wave Doppler to calculate early (E) and late (atrial, A) peak filling blood flow velocities and E-wave deceleration time. The E/A ratio represents the ratio of E wave to A wave. The sample volume was positioned at the tips of mitral valve leaflet in the mitral valve annulus, the ultrasound beam was in parallel with the direction of blood flow to record maximal transmitral flow velocities. Left atrial area was measured in apical four-chamber views, borders of the left atrium were traced just before mitral valve opening at end ventricular systole; examples for delineation of left atrial area in a 15-week-old K/BxN F1 and an age-matched KRN mouse are shown in Video S6 and Video S7, respectively. Parameters measured from M-mode echocardiography included interventricular septum thickness and left ventricle internal dimension in systolic and diastolic phase. Percentage ejection fraction was calculated from the M-mode measurements in the parasternal short axis view at the level of the papillary muscles. Cardiac function in hrAnxA1 treatment studies was assessed blindly.

Assessment of pericardial and pleural effusion *in vivo*

Long axis view of the heart was visualized using M-mode echocardiography. Pericardial effusion was visualized as an anechoic area between the pericardium and epicardium. Left and right side pulmonary fields were longitudinally scanned to visualize the pleural line, pleural space, and the lung. Pleural effusion was defined as an anechoic area between the lung and the chest wall (2).

Assessment of arthritic scores

Disease was monitored by assessing the clinical score; maximum 12 points per animal, 3 per limb with the following scoring system: 0, no evidence of inflammation; 1, inflammation in one of the following aspects: individual phalanges, localized wrist/ankle, or swelling on surface of paw; 2, inflammation on two aspects of paw; 3, major swelling on all aspects of paw (3). Arthritic scores in the studies where mice received hrAnxA1 were assessed blindly.

Quantification of cardiomyocyte size and collagen deposition

Hearts were collected and residual blood cleared by retrograde perfusion with 5 ml ice cold normal saline. Hearts were fixed in 4% paraformaldehyde solution for 24 h, then dehydrated in alcohol, embedded in paraffin and cut into 4-µm thick serial slides. The slides were deparaffinized with histoclear and rehydrated through graded alcohols to distilled water, stained with hematoxylin-eosin or collagen specific Sirius red, and viewed with a NanoZoomer Digital Pathology Scanner. Fifty random cardiomyocytes per hematoxylin-eosin section with a short-axis orientation (circular shape) and visible cell nucleus were selected for measurement of diameters using ImageJ analysis. Five random images per Sirius red-stained section were taken and quantification of Sirius red was performed using ImageJ. Cardiomyocyte size and collagen deposition in hrAnxA1 treatment study were assessed blindly.

Quantification of galectin-3 positive cells

In the time-course study, hearts were perfused as above and fixed in 4% paraformaldehyde solution for 24 h, then transferred to 15% sucrose in PBS until tissue sank, followed by 30% sucrose in PBS until tissue sank again. Hearts were then embedded in OCT and cut into 4-µm thick serial sections. Prior to staining, sections were left to dry for 30mins then permeabilized using TBS-T (Tris buffered saline with 0.01% Tween 20) for 15min at room temperature. Sections were blocked for 1h (Dako protein block) then incubated with goat anti-mouse gal-3 (polyclonal, R&D systems) in Dako antibody diluent. After washing 3 times with TBS-T, sections were incubated with donkey anti-goat Alexa fluor 594 (Thermo Fisher, Dartford, UK) for 1h in the dark at room temperature. Sections were then washed three times and mounted with fluoroshield™ containing DAPI (Sigma). Images were acquired on the NanoZoomer Digital Pathology Scanner. Quantification of Gal-3 positive cells was done using 20x magnification at 6 points across the left ventricle of each heart section, average Gal-3 positive cell counts of the 6 points taken from each mouse were used for comparison. Using ImageJ, the channels were split, and the red channel was used to quantify the number of Gal-3 positive cells for each heart. In the hrAnxA1 therapeutic study from week 8 to week 15, the staining was performed on formalin-fixed paraffin-embedded sections. Quantification of galectin-3 positive cells in the hrAnxA1 treatment study was assessed blindly.

Quantification of fluorescence intensity density of AnxA1 staining

Hearts were perfused as above and were fixed in 4% paraformaldehyde solution for 24 h, then dehydrated in alcohol, embedded in paraffin and cut into 4-µm thick serial sections. The slides were deparaffinized with histoclear and rehydrated through graded alcohols to distilled water. Slides were then transferred to TBS-T (Tris buffered saline with 0.01% Tween 20) for 15min at room temperature and then subjected to antigen retrieval with sodium citrate buffer (10mM citric acid, 0.05% Tween 20, pH 6.0) for 45min at 95°C. Slides were left to cool then rinsed with TBS-T and blocked for 1h (Dako protein block). Sections were then incubated overnight with rabbit anti-mouse AnxA1 (polyclonal, Abcam) in Dako antibody diluent. After washing 3 times with TBS-T, sections were incubated with goat anti-rabbit Cy3 (Thermo Fisher, Dartford, UK) for 1h in the dark at room temperature. Sections were then washed three times and mounted with fluoroshield™ containing DAPI (Sigma). Images were acquired on the NanoZoomer Digital Pathology Scanner. AnxA1 expression was quantified using ImageJ software using 20x magnification at 6 points across the left ventricle of each heart section. Images were split into their individual red/blue/green channels, the green channel was selected, and the same background threshold was applied to all images before quantification of fluorescence.

Flow cytometry

Hearts were perfused as above and cut into 1-mm^2 pieces, digested in 1 ml Hanks medium,

containing 608U/ml Collagenase I, 187.5 U/ml Collagenase XI, 90 U/ml Hyaluronidase and 90 U/ml DNase, for 1h at 37ºC with agitation as described (4). Digestion was quenched and filtered through 70 um nylon filter (BD Biosciences, San Jose, US) using 3 ml FACS buffer ($PBS^{-/-}$ buffer containing 0.02% bovine serum albumin). Cells were then centrifuged at 1,200 rpm for 10 min at 4 ºC, and were re-suspend in 500 µl FACS buffer for staining. Immune cells were differentiated using anti-CD45 (clone 30-F11; BioLegend, London, UK), anti-CD11b (clone M1/70; eBioscience, Dartford, UK), anti-F4/80 (clone BM8; BioLegend), anti-Ly6G (clone 1A8; BioLegend), anti-I-A/I-E (clone M5/114.15.2; BioLegend), anti-MerTK (clone 2B10C42, Biolegend), anti-CD206 (clone C068C2; Biolegend), anti-CD115 (clone AF598; Biolegend), anti-Siglec-F (clone E50-2440; BD Bioscience), anti-CD4 (clone GK1.5; eBioscience), anti-CD8 (clone 53-6.7; Biolegend) antibodies. In a separate set of experiment, fibroblasts were differentiated using anti-Thy1.2 (clone 30-H12; Biolegend), lineage antibody mixture (CD3e, CD11b, CD45R, Ly-6C, Ly-6G, and Ly76; Biolegend), anti-CD34 (clone HM34; Biolegend), anti-CD45 (clone 30-F11; Biolegend), anti-troponin T (clone 13-11; BD Bioscience), anti-CD31 (clone 390; BioLegend) antibodies (5). Surface expression of vascular cell adhesion molecule-1 (VCAM-1) was detected using anti-VCAM-1 antibody (clone 429 (MVCAM.A); BioLegend). Fixable Viability Dye eFluor™ 780 (eBioscience) was used to identify live cells. Cell counts were determined using Precision Count Beads (Biolegend). 10,000 live non-myocyte (NMC) events were acquired for fibroblast analysis, and 10,000 live CD45 events were acquired for macrophage analysis with a FACSCalibur (BD Biosciences) and analyzed using FlowJo analysis software (version 9.2, Treestar Inc., Ashland, US).

Surface expression of FPR2/ALX was determined on neutrophils, monocytes and lymphocytes in the blood of K/BxN F1 mice treated with hrAnxA1 or vehicle using primary rabbit anti-mouse FPR2/ALX antibody (Novus Biologicals, Centennial, US) and secondary anti-rabbit IgG conjugated with Alexa Fluor 488 (Thermo Fisher). Subtypes of white blood cells in the blood were differentiated using anti-Ly6G (clone 1A8; BioLegend), anti-Ly6C (clone HK1.4; BioLegend) and anti-CD3e (clone 145-2C11; eBioscience). Red blood cells lysis was carried out using whole blood lysing reagents (Bechman Coulter, Wycombe, UK) according to the manufacturer's instructions.

All flow cytometry analyses were carried out blindly.

Quantification of lung wet to dry weight ratio

Lung lobes were weighed (wet weight) on excision from the mice and then dried on hot blocks (55°C) for 48 hours, then reweighed (dry weight) (6).

Measurement of blood pressure and heart rate

Mice were anesthetized i.p. with 1.5 ml/kg of a ketamine (100 mg/ml)/xylazine (20 mg/ml) solution in a 2:1 ratio. Body temperature was maintained at 37±1°C by a homeothermic blanket unit (Harvard Apparatus Ltd, Edenbridge, Kent, UK). The right carotid artery was cannulated and connected to a pressure transducer (Senso-Nor 844, Horten, Norway), to continuously monitor mean arterial blood pressure, systolic pressure, diastolic pressure and heart rate (LabChart software, ADInstruments) for 10 minutes. Blood pressure and heart rate values of each mouse were calculated as the average of the data taken at the beginning of every minute during the 10-minute recording.

RT-PCR RNA quantification

The mRNA expression of exemplar pro-inflammatory, pro-resolving, fibrosis and hypertrophy markers were determined in whole mouse heart or kidney tissue. mRNA was extracted with

RNeasy Fibrous Tissue Mini Kit (Qiagen, Manchester, UK), and total RNA concentration and quality was determined with a ND-1000 spectrophotometer (Nano Drop Technologies, Wilmington, DE, USA). cDNA was synthesized from 1,000 ng RNA using the SuperScript™ VILO™ cDNA Synthesis Kit (Invitrogen, Dartford, UK) according to the manufacturer's instructions. Real-time quantitative PCR was performed using SYBR green ROX mix (Thermo Fisher) in the StepOnePlus™ thermal cycler (Applied Biosystems, Dartford, UK). Primers were purchased from Qiagen (*Gapdh, Hprt1, Il1b, Il1rn, Il6, Tnfa, Il10, Tgfb, Il4, Fpr2, Pomc, Lgals1, Lgals3, Lgals9, Havcr2, Ptgs1, Ptges, Alox5, Alox12, Alox15, Acta2, Nppa, Nppb, Timp1, Myh7, Col1a1, Col1a2, Col2a1, Col3a1, Mmp1a, Vegfa, Ifg1, Hgf1, Fgf2, Ly6g, Epx, Csf1r, Nos2, Hb2ab1, Arg1, Mc1r, Mc3r, Cd163, Scarb1*). Each sample was measured in duplicate. Cycle threshold values were determined by the StepOne software. Ct values were normalized using *Hprt1* as housekeeping gene and fold change of ΔCT was calculated relative to age-matched KRN controls. All RT-PCR analysis was conducted blindly.

Quantification of renal function

Renal function was assessed in 15 weeks old K/BxN F1 and age-matched KRN mice. Mice were euthanized with CO_2. Approximately 0.7 ml of blood was collected by cardiac puncture and centrifuged at 9900 *g* for 3 minutes to separate plasma. Plasma creatinine was analyzed blindly by MRC Harwell the clinical pathology service laboratory (Oxford, UK).

Figure S1

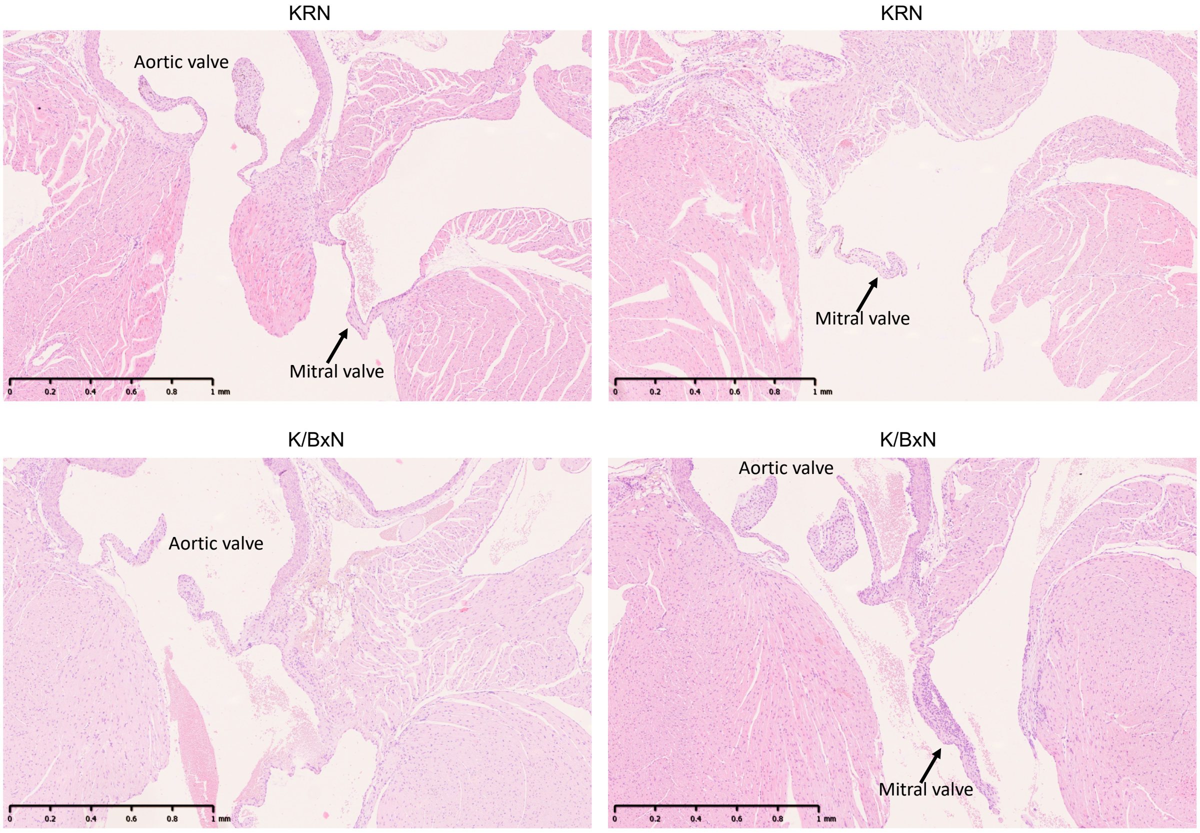

Figure S1. **Arthritic K/BxN F1 mice display mitral valve inflammation at week 15 as assessed by haematoxylin-eosin staining.** No overt aortic valve inflammation was observed in K/BxN F1 mice, but mitral valve inflammation was detected indicated by thickened mitral valve leaflets (arrows) in arthritic hearts; scale bars, 500 µm. K/BxN: K/BxN F1.

Figure S2

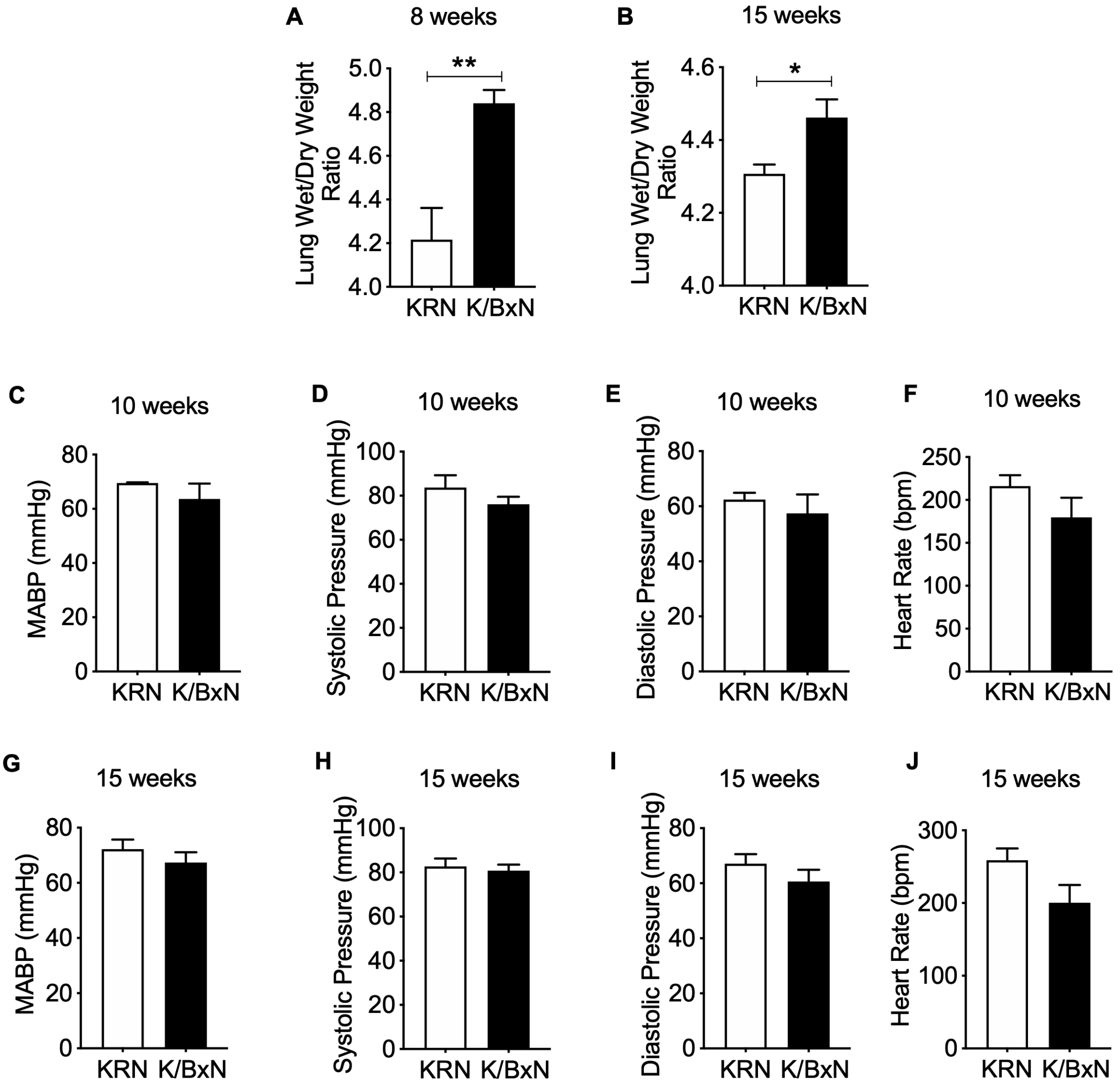

Figure S2. Increased lung wet to dry weight ratio and unaltered blood pressure and heart rate in K/BxN F1 mice compared with age-matched KRN mice. (**A**-**B**) Data are mean ± SEM of 4-6 mice per group. *$P<0.05$, **$P<0.01$ *vs.* non-arthritic KRN group. (**C**-**F**) Data are mean ± SEM of 3 mice per group. (**G**-**J**) Data are mean ± SEM of 8-12 mice per group. Data were analyzed by unpaired Student's t test. K/BxN: K/BxN F1; MABP: mean arterial blood pressure.

Figure S3

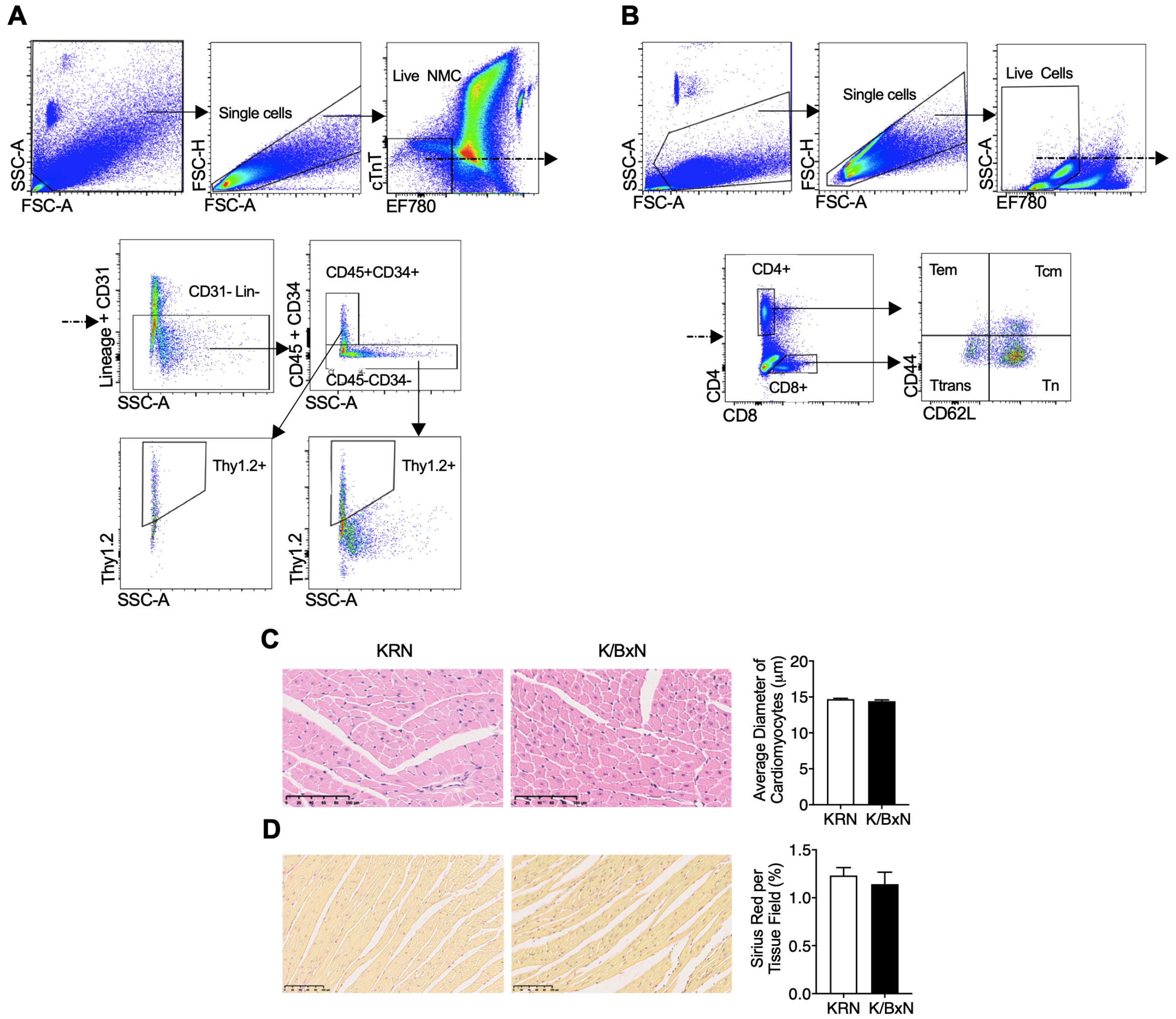

Figure S3. Flow cytometry gating strategy for fibroblasts and T cells; Arthritic K/BxN F1 mice at week-8 display normal average diameter of cardiomyocytes and collagen deposition as assessed by histology. (**A**) Flow cytometry gating strategy for fibroblasts in the mouse heart (week-8). Following selection of single cells, live non-muscle cells (NMCs) were separated using cardiac troponin T (cTnT) and viability dye EF780. CD31+Lineage+ cells were excluded and CD31-Lineage- cells were further separated using CD45 and CD34. CD45-CD34-Thy1.2+ cells were identified as structural fibroblasts, and CD45+CD34+Thy1.2+ monocytic fibroblasts. (**B**) Flow cytometry gating strategy for T cells in the mouse spleen (organ was chosen due to T cell abundancy; same gating strategy was applied to the heart). (**C**) Representative LV sections stained with haematoxylin-eosin and (**D**) Sirius red from arthritic and non-arthritic hearts and respective cumulative data; scale bars, 100 µm. KRN: n=4; K/BxN F1: n=5. Data are mean ± SEM and were analysed by unpaired Student's t test. K/BxN: K/BxN F1.

Figure S4

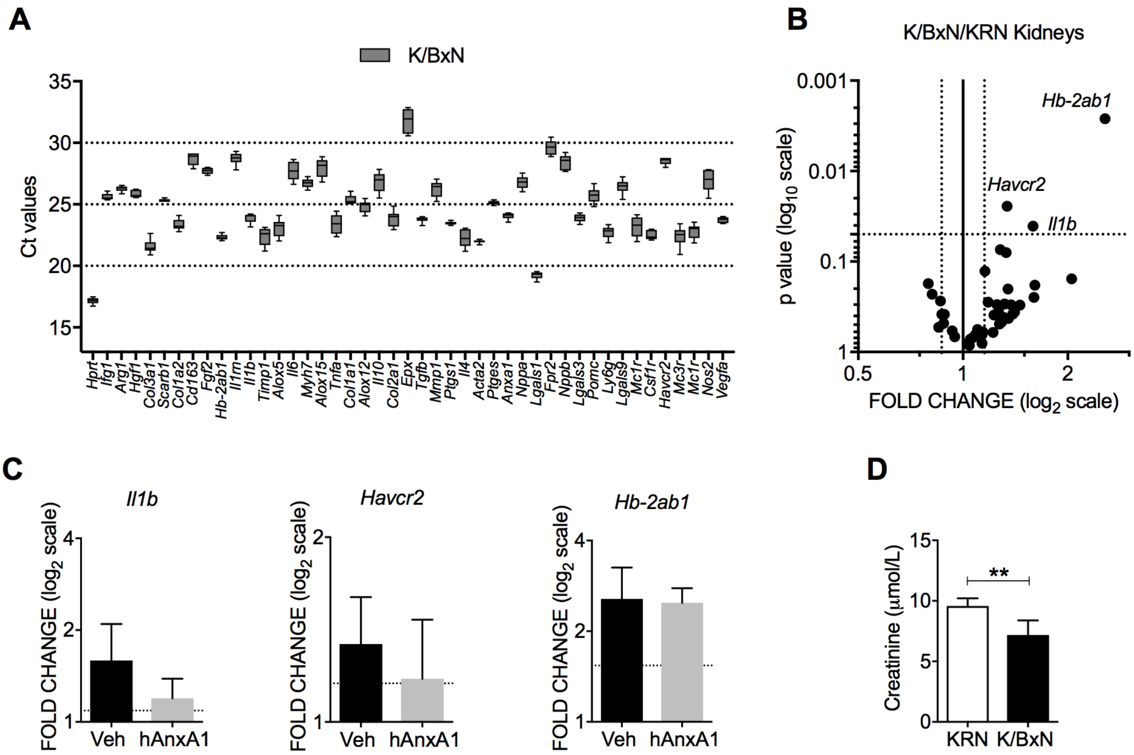

Figure S4. K/BxN F1 mouse kidneys shows limited mRNA expression modulation at week 8 and normal renal function at week-15. (**A**) Ct values of different genes analyzed by qPCR in K/BxN F1 kidneys at week 8. (**B**) Differential gene expression in K/BxN F1 mouse kidneys at week 8. Ct values were normalized using *Hprt1* as housekeeping gene and fold change was calculated relative to age-matched KRN controls. X-axis shows fold change (in $\log_2$ scale), with continued line (X=1) showing KRN mean and doted lines ± average SD of replicated PCR measures. Y-axis shows the p-value (in $\log_{10}$ scale) from t-test statistical analysis, with dotted line at P=0.05; n=4-6/group. (**C**) mRNA expression of *Il1b*, *Havcr2* and *Hb-2ab1* that were significantly upregulated in K/BxN F1 kidneys were not modulated by hrAnxA1 treatment. Ct values were normalized using *Hprt1* as housekeeping gene. Data are mean ± SEM of 4-6 mice per group, fold change was calculated relative to age-matched non-arthritic KRN controls (mean value=1), with dotted line showing ± average SD of replicated PCR measures for each gene. KRN group: n=4; K/BxN F1 + vehicle group: n=6; K/BxN F1 + hrAnxA1 group: n=6. (**D**) Renal function indicated by creatinine levels in 15-week old K/BxN F1 mice. Data are mean ± SEM. KRN group: n=4; K/BxN F1 group: n=5, **P<0.01 *vs.* non-arthritic KRN group. Data were analyzed by unpaired Student's t test. K/BxN: K/BxN F1; Veh: vehicle; hAnxA1: hrAnxA1.

Figure S5

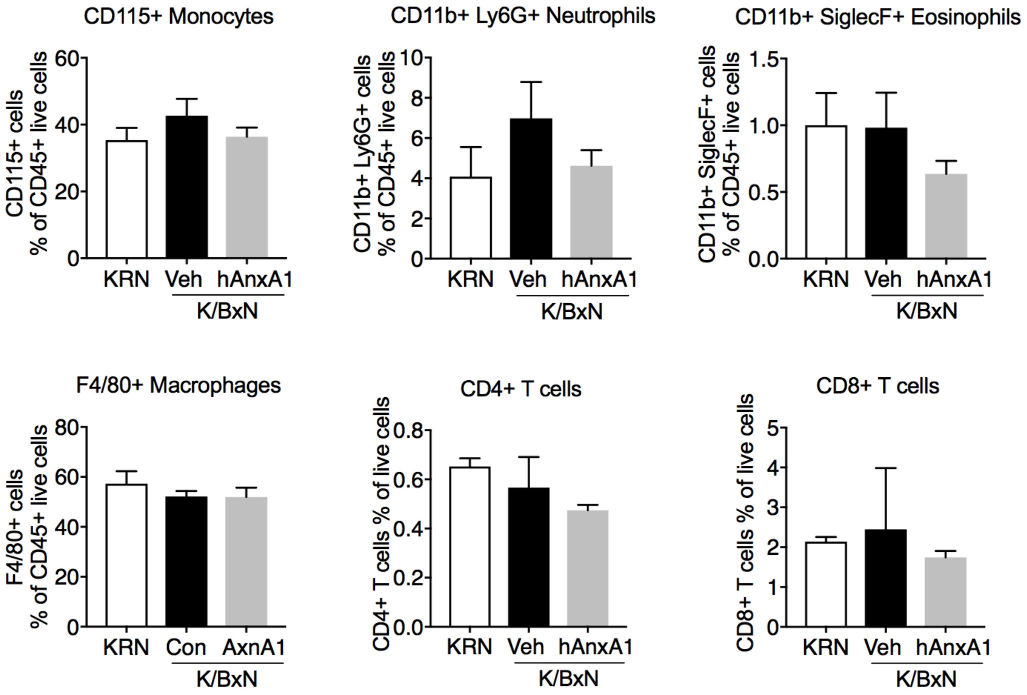

Figure S5. Major immune cell types in the heart as analyzed by flow cytometry at week 8. Cumulative data for cardiac CD115^{+} monocytes, CD11b^{+}Ly6G^{+} neutrophils, CD11b^{+} SiglecF^{+} eosinophils, F4/80^{+} macrophages, CD4^{+} and CD8^{+} T cells in non-arthritic KRN, arthritic K/BxN F1 and K/BxN F1 mice following treatment with hrAnxA1 (1 µg/mouse daily, from week 4 to week 8).

Data are mean ± SEM of n=4-5 per group, and were analyzed by one-way ANOVA followed by Bonferroni's test. Veh: vehicle; hAnxA1: hrAnxA1; Veh: vehicle; K/BxN: K/BxN F1.

Figure S6

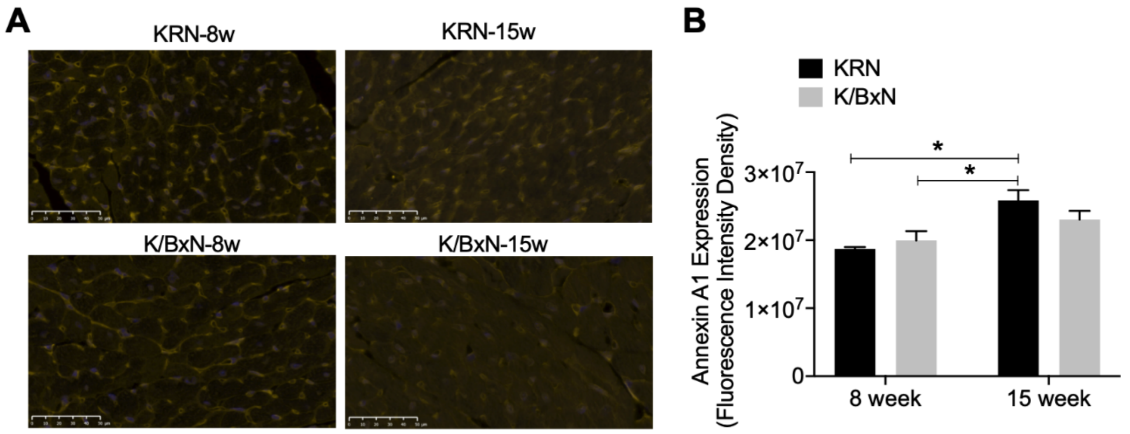

Figure S6. AnxA1 protein increases in KRN hearts over time but not in arthritic hearts. (A) Representative images of immunofluorescence; scale bars, 50 μm. **(B)** Quantification of fluorescence intensity density of AnxA1 staining in LV. n=4-6 per group. *P<0.05 vs. 15-week old KRN group (two-way ANOVA followed by Bonferroni's test). K/BxN: K/BxN F1.

Figure S7

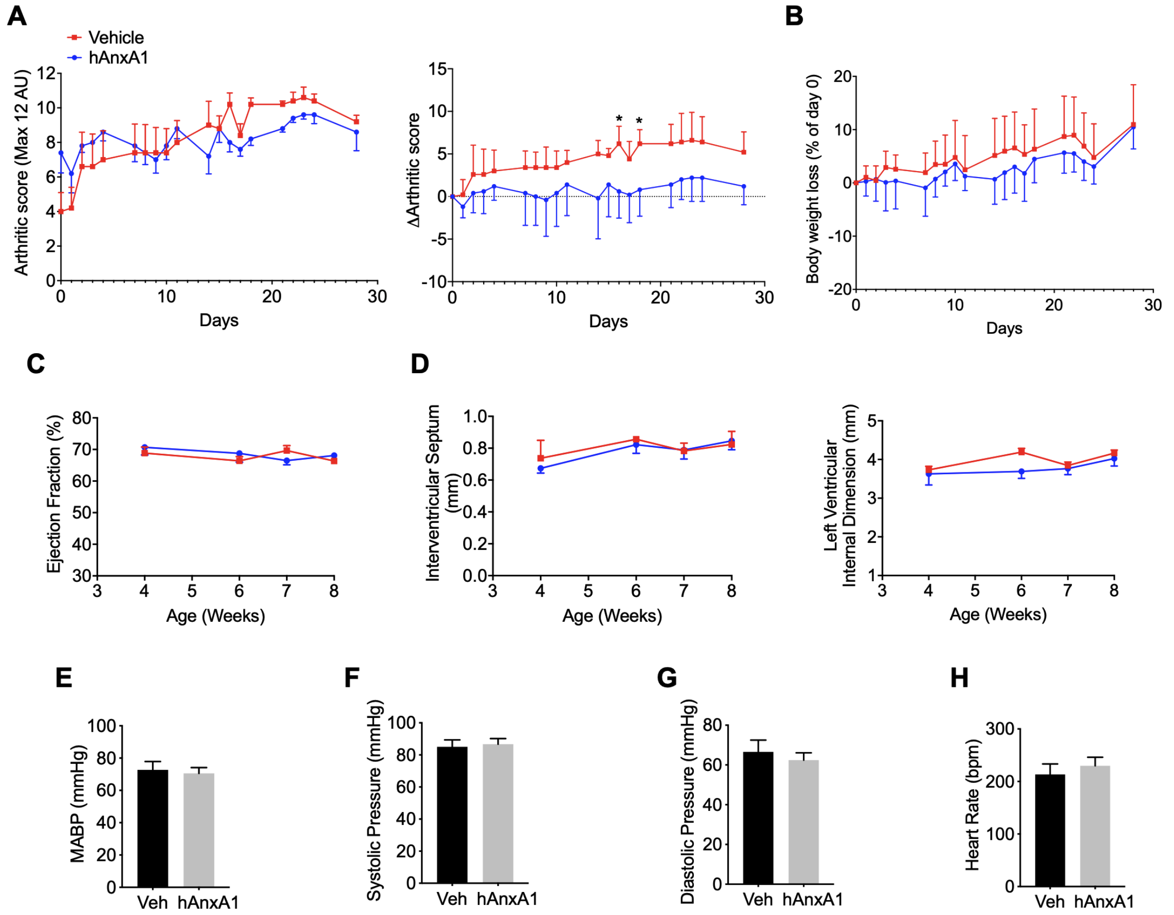

Figure S7. hrAnxA1 (week-4 to week-8) has moderate effects on arthritis and does not impact on body weight loss, LV size or systolic function in K/BxN F1 mice. (**A**) Clinical severity of arthritis was assessed visually using a defined severity scoring system (max score 12) in K/BxN F1 mice receiving vehicle or hrAnxA1. ΔArthritic score represents changes of arthritic score over baseline score at day 0 within each group. (**B**) Body weight loss expressed as a percentage of weight at day 0. No significant alterations in EF (**C**), interventricular septum thickness or LV internal diastolic dimensions (**D**) was observed in hrAnxA1-treated arthritic mice. Mean arterial blood pressure (**E**), systolic pressure (**F**), diastolic pressure (**G**), and heart rate (**H**) were not affected by hrAnxA1 treatment (week 7 to week 8, 1μg s.c. daily) in arthritic mice. (**A-D**) Data are mean ± SEM. K/BxN F1 + vehicle group: n=5; K/BxN F1+ hrAnxA1 group: n=5. *P<0.05 *vs.* vehicle group (two-way ANOVA followed by Bonferroni's test). (**E-H**) Data are mean ± SEM. K/BxN F1 + vehicle group: n=4; K/BxN F1+ hrAnxA1 group: n=5. Data were analyzed by unpaired Student's t test. MABP: mean arterial blood pressure; Veh: vehicle; hAnxA1: hrAnxA1.

Figure S8

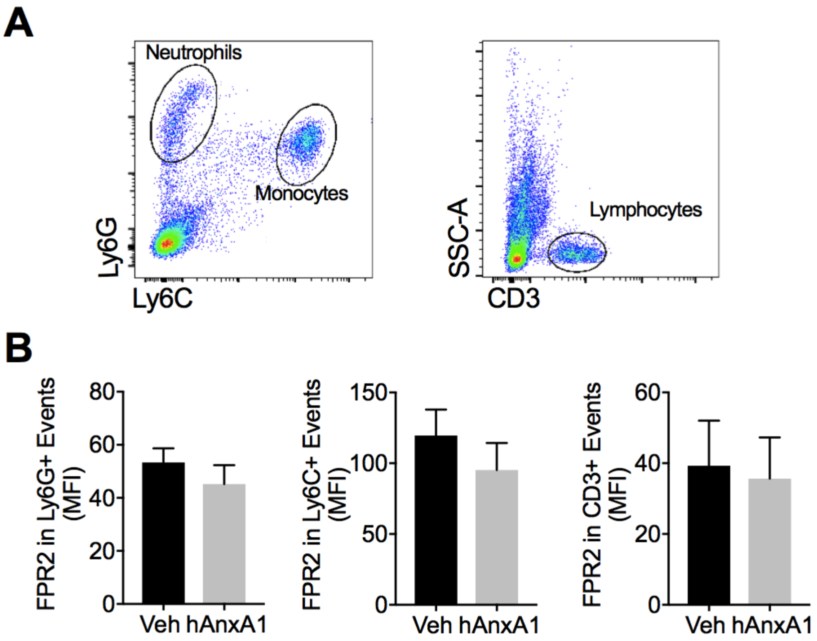

Figure S8. hrAnxA1 treatment (week-4 to week-8) did not alter surface FPR2 expression in blood immune cells of K/BxN F1 mice. (**A**) Scattergrams illustrating blood Ly6G$^+$ neutrophil, Ly6C$^+$ monocyte and CD3$^+$ lymphocyte positive events. (**B**) Cumulative data show FPR2 median fluorescence intensity (MFI) in blood Ly6G$^+$ neutrophils, Ly6C$^+$ monocytes and CD3$^+$ lymphocytes in K/BxN F1 mice receiving vehicle or hrAnxA1 treatment. K/BxN F1 + vehicle group: n=3; K/BxN F1 + hrAnxA1 group: n=4. Data are mean ± SEM and were analyzed by unpaired Student's t test. Veh: vehicle; hAnxA1: hrAnxA1.

Figure S9

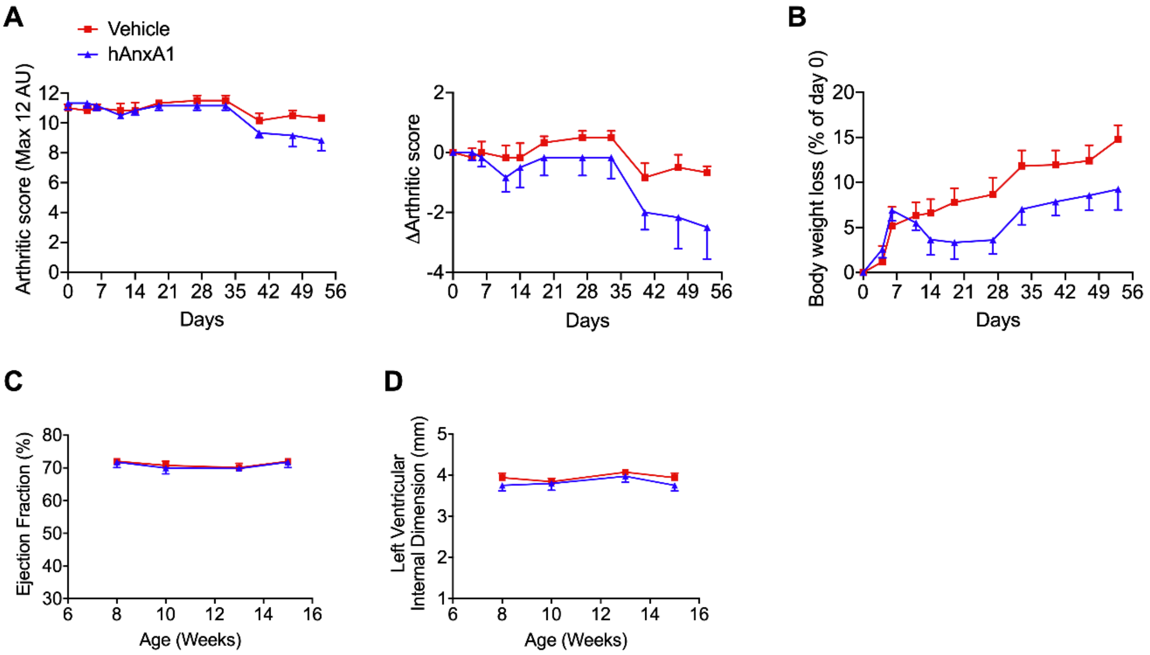

Figure S9. hrAnxA1 (week-8 to week-15) has moderate effects on arthritis and does not impact on body weight loss or LV systolic function in K/BxN F1 mice. (**A**) Clinical severity of arthritis was assessed visually using a defined severity scoring system (max score 12) in K/BxN F1 mice receiving vehicle or hrAnxA1. ΔArthritic score represents changes of arthritic score over baseline score at day 0 within each group. (**B**) Body weight loss expressed as a percentage of weight at day 0. No significant alterations in EF (**C**) or LV internal diastolic dimensions (**D**) was observed in hrAnxA1-treated arthritic mice.

Data are mean ± SEM and were analysed by two-way ANOVA followed by Bonferroni’s test. K/BxN F1 + vehicle group: n=6; K/BxN F1+ hrAnxA1 group: n=6. hAnxA1: hrAnxA1.

Supplementary Videos

Video S1. **Representative color Doppler of the mitral valve shows that a 15-week-old K/BxN F1 mouse developed trivial mitral regurgitation**. This is indicated by a blue jet area in LA as mitral valve closes at the early stage of systolic phase.

Video S2. **Representative color Doppler of the mitral valve shows that a 15-week-old K/BxN F1 mouse did not show signs of mitral regurgitation**. No obvious mitral stenosis was observed as indicated by similar pattern/width of color jet in LV during diastolic phase to that observed in age-matched KRN mice (as shown in Video S3).

Video S3. **Representative color Doppler of the mitral valve shows that a 15-week-old KRN mouse developed trivial mitral regurgitation**. This is indicated by a blue jet area in LA as mitral valve closes at the early stage of systolic phase.

Video S4. Absence of pericardial effusion in 15-week-old K/BxN F1 mice. Long axis view of the heart was visualized using M-mode echocardiography. No obvious anechoic area between the pericardium and the epicardium was observed, thus the LV diastolic dysfunction in K/BxN F1 mice was not secondary to pericardial effusion.

Video S5. **Absence of pleural effusion in 15-week-old K/BxN F1 mice**. Left and right side pulmonary fields were longitudinally scanned to visualize the pleural line, pleural space, and lung layers. No obvious anechoic area between the lung and the chest wall including the space closed to the liver was observed, thus there was no pleural effusion in K/BxN F1 mice.

Video S6. **Example for delineation of left atrial area in a 15-week-old K/BxN F1 mouse**.

Video S7. **Example for delineation of left atrial area in a 15-week-old KRN mouse**.

References

1. G. S. D. Purvis *et al.*, Annexin A1 attenuates microvascular complications through restoration of Akt signalling in a murine model of type 1 diabetes. *Diabetologia* **61**, 482-495 (2018).
2. M. Villalba-Orero *et al.*, Lung ultrasound as a translational approach for non-invasive assessment of heart failure with reduced or preserved ejection fraction in mice. *Cardiovasc Res* **113**, 1113-1123 (2017).
3. L. V. Norling *et al.*, Proresolving and cartilage-protective actions of resolvin D1 in inflammatory arthritis. *JCI Insight* **1**, e85922 (2016).
4. I. Komarowska *et al.*, Hepatocyte Growth Factor Receptor c-Met Instructs T Cell Cardiotropism and Promotes T Cell Migration to the Heart via Autocrine Chemokine Release. *Immunity* **42**, 1087-1099 (2015).
5. P. Sirish *et al.*, Unique mechanistic insights into the beneficial effects of soluble epoxide hydrolase inhibitors in the prevention of cardiac fibrosis. *Proc Natl Acad Sci U S A* **110**, 5618-5623 (2013).
6. J. C. Parker, M. I. Townsley, Evaluation of lung injury in rats and mice. *Am J Physiol Lung Cell Mol Physiol* **286**, L231-246 (2004).
